# Sponge-like Cryogels from Liquid–Liquid Phase Separation: Structure, Porosity, and Diffusional Gel Properties

**DOI:** 10.1021/acsami.3c03239

**Published:** 2023-07-29

**Authors:** Rosangela Mastrangelo, Claudio Resta, Emiliano Carretti, Emiliano Fratini, Piero Baglioni

**Affiliations:** Department of Chemistry and CSGI, University of Florence, via della Lastruccia, 3, Sesto Fiorentino, Florence 50019, Italy

**Keywords:** poly(vinyl alcohol) cryogels, porogens, liquid−liquid phase separation, gelation mechanism, tortuosity, art conservation, cleaning

## Abstract

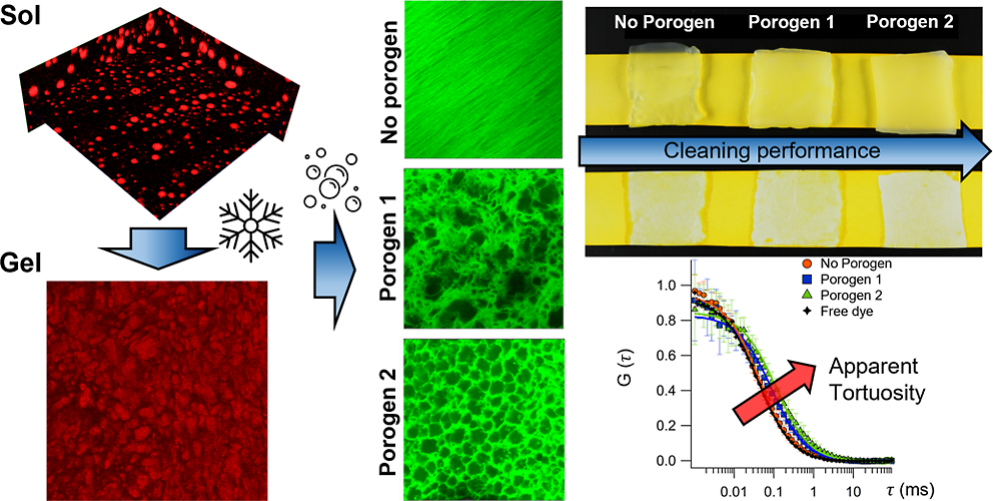

Macroporous gels find application in several scientific fields, ranging from art restoration to wastewater filtration or cell entrapment. In this work, two-component sponge-like cryogels are challenged to assess their cleaning performances and to investigate how pores size and connectivity affect physico-chemical properties. The gels were obtained through a freeze–thaw process, exploiting a spontaneous polymer–polymer phase-separation occurring in the pre-gel solution. During the freezing step, a highly hydrolyzed polyvinyl alcohol (H-PVA) forms the hydrogel walls. The secondary components, namely a partially hydrolyzed polyvinyl alcohol (L-PVA) or polyvinyl pyrrolidone (PVP), act as modular porogens, being partially extracted during gel washing. H-PVA/L-PVA and H-PVA/PVP mixtures were studied by confocal laser scanning microscopy to unveil sols and gels morphology at the micron-scale, while small angle X-ray scattering was used to get insights about characteristic dimensions at the nanoscale. The gelation mechanism was investigated through rheology measurements, and the characteristic exponents were compared to De Gennes’ scaling laws gathered from percolation. In the field of art conservation, these sponge-like gels are ideal systems for the cleaning of artistic painted surfaces. Their interconnected pores allow the diffusion of cleaning fluids at the painted interface, facilitating dirt uptake and/or detachment. This study uncovered a direct relationship linking a gel’s cleaning performance to its apparent tortuosity. These findings can pave the way to fine-tuning systems with enhanced cleaning abilities, not restricted to the restoration of irreplaceable priceless works of art, but with possible application in diverse research fields.

## Introduction

Pores size and connectivity are two of the key-features of functional hydrogels:^1^ their study is fundamental to get insights on the gel topology at different length scales and to link their structural features to transport properties.^2^ Conventional chemical hydrogels typically show, considering the average distance between crosslinks, a few nanometers of pore size^3^ limiting the application in fields like bio-adhesives, tissue engineering,^4^ bioseparation,^5^ and sorbents,^6^ where specific pores size ranges and transport properties are required. Hydrogels featuring tailored micron-sized pores can address this gap, finding application in stimuli-responsive gels for drug delivery,^7^ filtration of wastewater with exclusion of particulate and pathogens,^8−10^ cell growth, adhesion, and entrapment.^11,12^ To increase gels pores size, different approaches can be followed. Solvent casting, freeze drying, gas foaming,^13−15^ and phase–phase separation are some of the processes that lead to macroporous gels.^1^ Phase-separation methods reported in the literature imply the use of immiscible organic solvents or hydrophobic polymers.^16,17^ In both cases, the properties of the final network are affected, while the final pore architecture is hard to control.^1^

Recently, we reported on the so called twin-chain gels,^18,19^ i.e., aqueous poly(vinyl alcohol) (PVA)-based systems that experienced a spontaneous polymer–polymer phase-separation at room temperature, leading to continuous and dispersed phases that can be “frozen” in a gel state, through a freeze–thaw (FT) process. The sponge-like morphology of the final gels can be tailored and controlled by choosing polymer pairs differing by molecular weight and hydrolysis degree, experiencing different phase-behavior. The FT process leads to the formation of a cryogel.^20,21^ Cryogels are known to be strong physical gels, obtained by the combination of spinodal decomposition and the polymer crystallization, occurring during the freezing of polymer solutions.^22−24^ The resulting networks are highly elastic and resilient, to the point of being considered good candidates in biomedical applications.^25^ PVA cryogels have been largely investigated in the literature,^26^ nonetheless, the ease with PVAs undergo phase separation, when mixed with hydrophilic polymers in aqueous solution, has taken a back seat and has never been fully explored.

PVA-based cryogels designed by our group^18,27−29^ were proven to be non-risky effective tools for the cleaning of rough, water-sensitive artistic substrates. High adaptability, interconnected porosity, high free water content, and water retentiveness are some of the key-features that granted these cryogels unprecedented cleaning performances on masterpieces by Jackson Pollock^18^ and Pablo Picasso,^19^ among many others. However, a deep understanding of gel transport properties and cleaning mechanism is still lacking.

In this work, the physico-chemical properties of PVA-based cryogels with a tailored, micron-sized porosity are systematically investigated. More specifically, the effects of two polymers acting as porogens, i.e., a partially hydrolyzed PVA, L-PVA, and poly(vinyl pyrrolidone), PVP, on the main H-PVA network, is reported to unveil the coupling between the gels’ structural features and their diffusional properties. Our ultimate goal is to find connections between pre-gel solutions and gels morphologies/structure, aiming at a rational design of sponge-like networks for targeted applications, not restricted to the restoration field.

The polymer–polymer phase-separation and the gels morphology were investigated through confocal laser scanning microscopy (CLSM) at 25 °C. Pores shape and size were proven to depend on the H-PVA/porogen polymer ratio and polymers behavior in aqueous solution. The effects of the phase-separation on gels structural features, such as crystallinity (obtained from differential scanning calorimetry, DSC), characteristic mesh-size at the nanoscale, and mechanical properties were assessed through small angle X-ray scattering (SAXS) and rheology measurements. Gels washing caused a significant or complete extraction of the porogens, quantified though nuclear magnetic resonance (NMR) measurements. Gels sub-micron sized pores were observed through scanning electron microscopy (SEM).

Overall, the phase-separation leads to networks with higher apparent tortuosity^30,31^ (obtained from fluorescence correlation spectroscopy, FCS, data) that, ultimately, affects gels transport properties and cleaning abilities. Counterintuitively, slower diffusion rates in sponge-like gels enhanced cleaning performances, suggesting that pores connectivity is the key to control “materials” uptake/delivery from an interface.

## Materials and Methods

### Chemicals

H-PVA (polyvinyl alcohol with hydrolysis degree, HD, 98% and *M*_w_ 160 kDa), L-PVA (polyvinyl alcohol with HD 88% and *M*_w_ 100 kDa), and PVP (polyvinyl pyrrolidone, *M*_w_ 1300 kDa) for the hydrogel preparation were purchased from Sigma-Aldrich.

Water for the hydrogels preparation was purified through a Millipore system (resistivity > 18 MΩ cm). The following fluorescent dyes were used in CLSM or FCS calibration/measurements: fluorescein isothiocyanate isomer I (FITC, purity ≥ 90%, Sigma-Aldrich); rhodamine B isothiocyanate (RBITC, mixed isomers, Sigma-Aldrich); rhodamine 110 chloride (purity ≥99%, Sigma-Aldrich); Alexa Fluor 568 (Thermo Fisher Scientific). The tartrazine (Dye content ≥ 85%, Sigma-Aldrich) was used in the dye uptake, diffusion kinetics, and cleaning test experiments. All the chemicals were used as received, without further purification.

### Cryogel Preparation

Gels are obtained through a FT process. Polymer mixtures were dissolved in purified water, in a rounded-bottom flask equipped with a condenser to prevent water evaporation. Temperature was set at 95 °C and the mixture maintained under continuous stirring for 2 h. After complete dissolution, pre-gel solutions were cooled down to room temperature (24 h), poured into polystyrene molds (14 × 7 × 0.2 cm^3^), and then frozen at −18 °C. Gels were obtained after 1 FT cycle. Different cryogels were obtained by varying the H-PVA concentration (*X*, in % w/v).

Gels made of H-PVA only are indicated throughout the paper as Pure Networks (PN_*X*), while gels also containing L-PVA or PVP are referred as i-PVA_*X* and i-PVP_*X*, respectively. i-PVA_*X* and i-PVP_*X* contain a variable amount of H-PVA (*X*, in % w/v) that is combined with a fixed amount (i.e., 3% w/v) of the porogen polymer (i.e., L-PVA or PVP). This notation has been selected in such a way that samples with the same *X* value contains the same amount of H_PVA. Further details can be found in the Supporting Information.

The list of the principal investigation techniques used in the present paper is reported below. Further information and the equations used to analyze the data can be found in the Supporting Information.

### Polymers Labeling

H-PVA and L-PVA were chemically labeled with FITC and RBITC, respectively. Details on both labeling reaction are reported elsewhere.^18^ PVP was labeled with RBITC, following the same procedure used for L-PVA. Additional details are reported in the Supporting Information.

### CLSM Imaging

CLSM experiments were performed on a Leica TCS SP8 confocal microscope (Leica Microsystems GmbH, Wetzlar, Germany), equipped with a 63X/1.2 Zeiss water immersion objective. The dyes rhodamine 110 (used to acquire images of the washed gels) and FITC (for H-PVA labeling) were excited with a 488 nm laser line (Ar laser) and the fluorescence emitted was collected by a photomultiplier tube (PMT) in the 498–540 nm range. RBITC (for L-PVA and PVP labeling) was excited with a 561 nm laser (DPSS 561) and the fluorescence was collected by a PMT in the 571–630 nm range. 3D stacks, containing 150–170 2D images, were acquired for pre-gel and gelled systems.

### Crystallinity Degree (*X*_c_)

The degree of crystallinity of the cryogels was evaluated through DSC measurements and, specifically, by integrating the melting peaks observed for the freeze-dried samples. *X*_c_ was calculated as the ratio between the specific enthalpies of fusion of the gel sample and a fully crystalline PVA (161 J/g),^32^ respectively. The DSC temperature ramp was set from 25 to 250 °C, at a heating rate of 5 °C/min. The reported *X*_c_ values, for both thawed and washed gels, are averages of 3 measurements.

### Gel Fraction (*G* %)

The fraction of polymer chains taking part in the gel structure can be evaluated gravimetrically. Namely, the percentage gel fraction was obtained as follows:
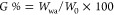
1

*W*_0_ is the initial polymer content in the gel, while *W*_wa_ is the polymer content of the same sample after washing. *W*_wa_ was obtained by oven drying washed gel samples at 100 °C, to a constant weight. Values reported are averages of at least three measurements.

### Equilibrium Volume Swelling Ratio (*q*_v_)

The swelling degree was evaluated on washed gel sheets. The swelling capacity was calculated by measuring the variation of the width by means of a caliper rule (resolution 0.02 mm) according to the following equation:^33^
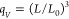
2where *L*_0_ is the side length after preparation (i.e., the mold’s side), and *L* is the side length after washing (at swelling equilibrium). *q*_v_ was not calculated in the case of PN_5 sample, as swelling in water caused some instability in the sample (i.e., breaking in some points).

### Small-Angle X-ray Scattering

SAXS curves were collected with a HECUS S3-MICRO SWAXS-camera (Hecus XRS, Graz, Austria), equipped with a Kratky collimation system and a position-sensitive detector (50 M), with 1024 channels (width = 54 μm). A Cu anode provided Cu Kα radiation (λ = 0.1542 nm) by using a 50 W microfocus source with customized FOX-3D single-bounce multilayer point focusing optics (GeniX system, Xenocs, Grenoble, France).

### Fluorescence Correlation Spectroscopy

A Leica TCS SP8 confocal microscope (Leica Microsystems GmbH, Wetzlar, Germany), equipped with a PicoQuant FCS modulus (PicoQuant, Berlin, Germany) with a 63X/1.2 Zeiss water immersion objective was used.

### Rheology

Rheology measurements were performed by using a Discovery HR-3 rheometer from TA Instruments (40 mm diameter parallel plate geometry), equipped with a Peltier temperature control system.

### Nuclear Magnetic Resonance

NMR experiments were performed on a Bruker Advance Spectrometer operating at the frequency of 400 MHz for ^1^H in DMSO-*d*_6_.

### Scanning Electron Microscopy

SEM images were collected through a field emission gun scanning electron microscope by SIGMA (Carl Zeiss Microscopy GmbH, Germany). The acceleration potential was set to 2 kV and the working distance to 3.4 mm. Gel samples were freeze-dried and images were collected on uncoated samples.

### Dye Uptake Experiments and Cleaning Tests

Cardboard sheets were soaked in demineralized water at first, then in a 2.5% w/w tartrazine aqueous solution (1 min) and air dried. Gel sheets were cut, gently dried on Whatman paper and placed on the dyed cardboard: the gel-paper interaction was recorded over a 10 min period, and pictures extracted from the movies were used to evaluate the dye solution rise through the gel matrices. Briefly, the rise was measured through ImageJ: images were zoomed and the contrast was enhanced to clearly observe the front position. The gel thickness reduction due to water evaporation (1–5% in all the analyzed samples) was evaluated. Pictures of the cardboard cleaned areas were taken right after gel removal. The dye removal was evaluated again with ImageJ: images were converted in grayscale, and the average grayscale intensity was calculated in 400 × 400 pixels^2^ squares, centered inside each cleaned area. Standard deviations were also obtained. Each cleaned area is about 800 × 760 pixels^2^ large. The grayscale values range between 0 (black) and 255 (white).

### Tartrazine Diffusion Kinetics

Diffusion of tartrazine across the gels sheets was monitored through a kinetic experiment, in which gels were kept in contact with an aqueous tartrazine solution. Briefly, gels were equilibrated in a 0.25% w/w tartrazine aqueous solution. Later, gels were used as membranes in homemade Franz diffusion cells: they were sealed between two quartz cuvettes, the first containing the 0.25% w/w tartrazine aqueous solution, the second containing milliQ water. All gels had 2 mm thickness. Tartrazine concentration in milliQ compartment was measured by monitoring the dye absorbance at λ_max_ = 427.5 nm for 17 h ca.

## Results and Discussion

### Cryogels Morphology in the Presence of L-PVA and PVP Porogens

Cryogels were prepared by combining a highly hydrolyzed PVA, H-PVA, to form the main gel network and L-PVA, a partially hydrolyzed PVA, or PVP. The investigated systems contained increasing quantities of H-PVA (*X*, % w/v) and a fixed L-PVA or PVP concentration (and indicated as i-PVA or i-PVP). Gels containing only H-PVA are labelled as PN (Pure Networks) and used as a reference system.

**Figure 1:**
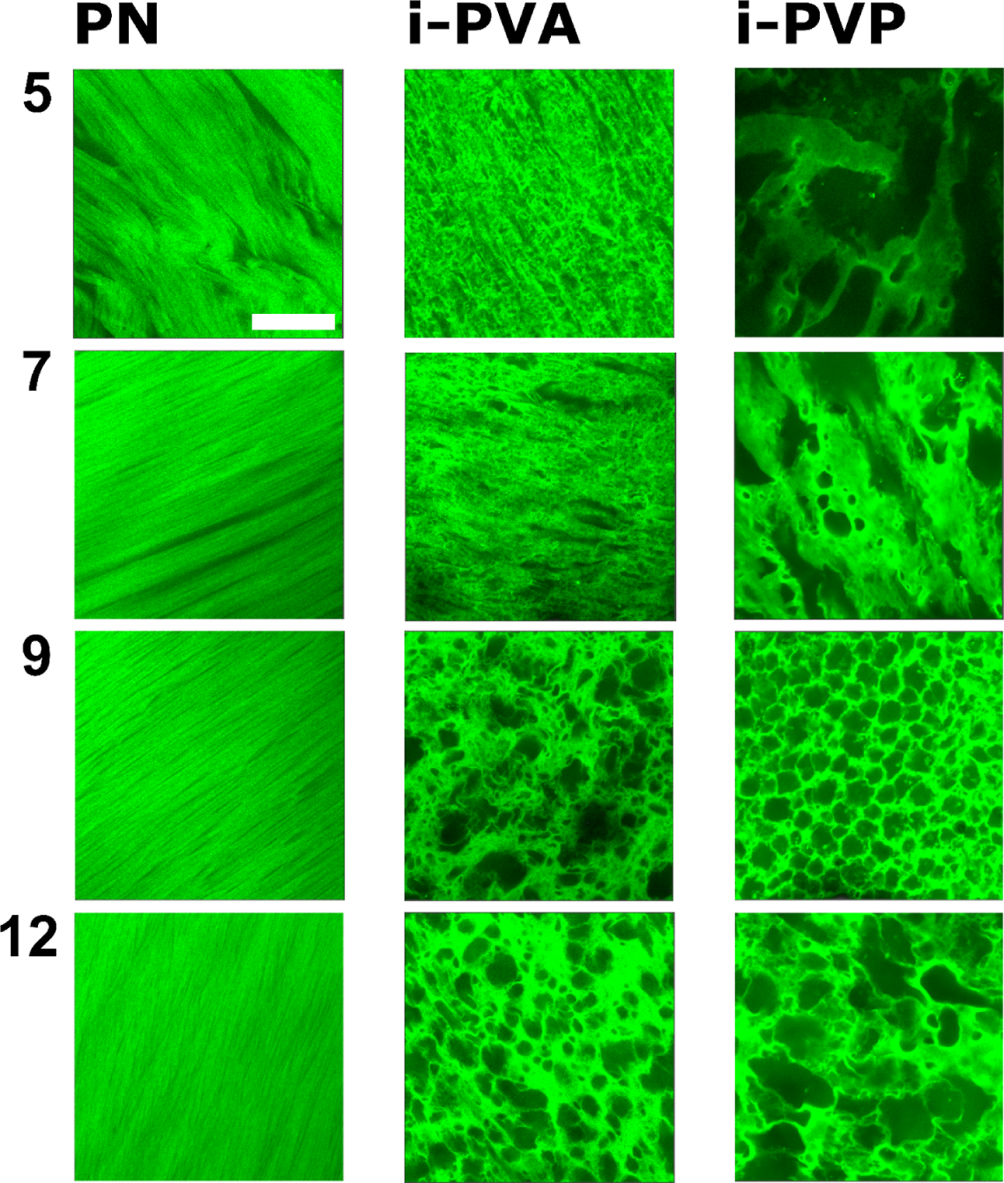
Confocal images of PN, i-PVA, and i-PVP gels for different H-PVA concentrations. *X* = 5, 7, 9, and 12 samples are shown in each row. Gels were washed in demineralized water (7 days) and then equilibrated in a rhodamine 110 solution. Scalebar: 50 μm.

Representative confocal images of PN_*X*, i-PVA_*X*, and i-PVP_*X* gels (H-PVA concentration *X* = 5, 7, 9,12% w/v), extracted from 3D confocal stacks (Figure S1), are shown in Figure 1. Images were acquired on gel samples after 1 week washing/storage in water. PN samples are strongly anisotropic, presenting an elongated, needle-shaped porosity that can be ascribed to the crystallization of ice in aligned needles, on surfaces perpendicular to the direction of freezing, i.e., on planes parallel to the gel sheet’s main surface.^18,34−36^ This results in an ordering of the gel structure, where straight gels strands are interspersed with elongated pores. Directionality, in the PN series, becomes more evident as the concentration of H-PVA increases (i.e., *X* ≥ 7). In i-PVA and i-PVP series, *X* = 5 and *X* = 7 gels show pores with elongated and random shapes, with a certain degree of directionality that is still visible. Samples with *X* = 9 and *X* = 12 present sponge-like structures, with pores that are almost spherical.

The gel nature of the samples, after FT and washing, can be inferred from Figure S2, showing the frequency sweeps, acquired at constant strain, of the investigated systems. More specifically, all gels show “a storage modulus, *G*′(ω), which exhibits a pronounced plateau extending to times at least of the order of seconds and a loss modulus, *G*″(ω), which is considerably smaller that the storage modulus in the plateau region”.^37^

**Figure 2:**
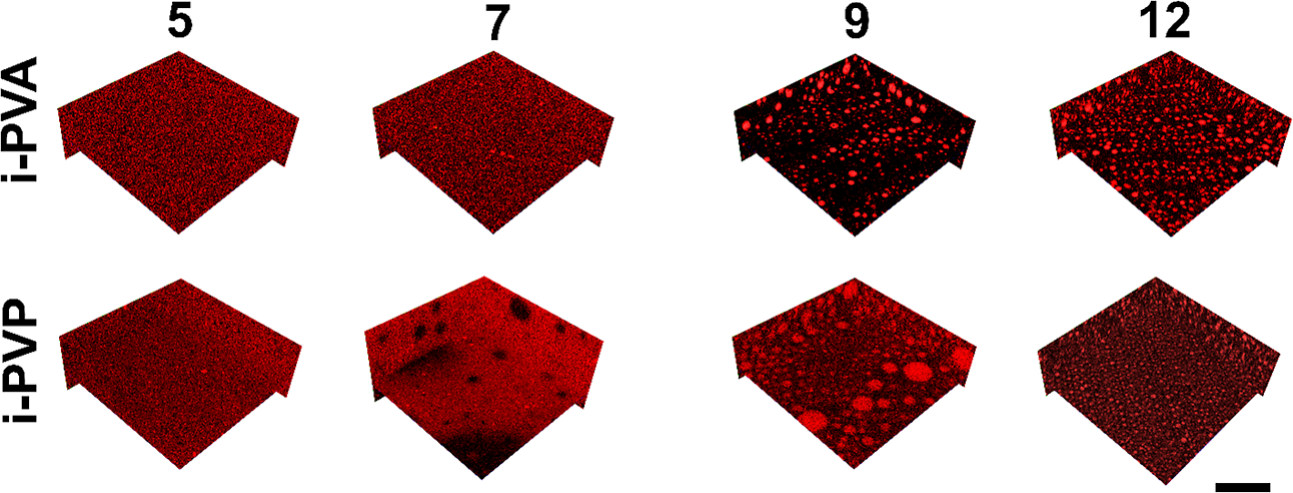
Confocal images of RBITC-labeled i-PVA_5-12 and i-PVP_5-12 pre-gel solutions at room temperature. In i-PVP_5 only the PVP-rich area is shown. Scalebar is 50 μm.

Figure 1 suggests that i-PVA_*X* and i-PVP_*X* cryostructuration occurred differently from PN gels. Pore shape and size agree with the morphology of the pre-gel solutions. In a recent work,^18^ we found that the structural changes in H-PVA cryogels, induced by the addition of L-PVA, are associated to the polymer–polymer segregation occurring in aqueous solution before freezing. In this case, L-PVA formed blobs acting as porogens during the gel cryoformation. Confocal images of i-PVP gels suggest that a similar phase-behavior might occur also for i-PVP pre-gel solutions. To confirm these hypotheses, morphologies of i-PVA and i-PVP pre-gel solutions were investigated through CLSM imaging, 24 h after preparation. Confocal images of pre-gel solutions, containing RBITC-labeled L-PVA and PVP are shown in Figure 2.

Phase-separation was observed in i-PVA_9 and i-PVA_12 solutions (Figure 2, i-PVA row, 9 and 12), where L-PVA concentrated in blobs. i-PVP_5 and i-PVP_7 (Figure 2, i-PVP row, 5 and 7) showed H-PVA-rich and PVP-rich areas. In i-PVP_5, the solution was characterized by large portions (100–200 μm) containing either H-PVA or PVP; a PVP-rich area is shown, specifically, in Figure 2. In i-PVP_7, some portions of the solution showed a “reverse” phase-separation, with PVP-rich areas containing H-PVA blobs (see black features in Figure 2, i-PVP row, 7). However, this behavior was not representative of the whole sample. Finally, spherical blobs were identified in i-PVP_9 and i-PVP_12 (Figure 2, i-PVP row, 9 and 12).

2D images of gels after thawing, extracted from 3D confocal stacks and containing RBITC-labeled L-PVA and PVP, are shown in Figure S3. During freezing, L-PVA and PVP blobs deformed and enlarged due to the stress of freezing water. After thawing, blob shape and size can be compared to those of gel pores, shown in Figure 1. An accurate control of gels porosity can be achieved only when pre-gel solutions are characterized by a disperse phase with homogenous, spherical blobs, i.e., in the case of *X* = 9 and *X* = 12 systems.

**Figure 3:**
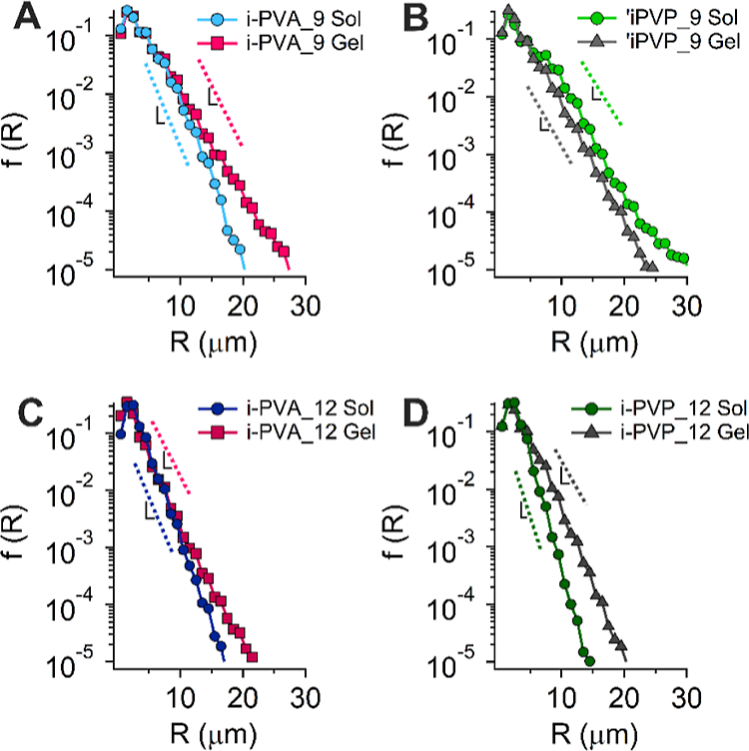
Chord length analysis of blobs in pre-gel solutions and cryo-formed gels after thawing; (A) i-PVA_9; (B) i-PVP_9; (C) i-PVA_12; (D) i-PVP_12. In all samples, except for i-PVP_9, blob size increases after the FT process.

The low temperature of the freezing step is another factor influencing blob size in pre-gel solutions, right before freezing. As the FT process occurs at −18 °C, the systems must reach sub-zero temperatures before water starts freezing. Such a low temperature is expected to enhance polymer–polymer demixing, resulting in a general increase of blob size. The change in blob size was quantified by applying the chord length distribution method^38^ to the 3D confocal images of *X* = 9, 12 pre-gel solutions, and gels after thawing (Figure 2C,D,G,H and Figure S1C,D,G,H, respectively). 100 2D images per sample were analyzed, and the averaged trends are shown in Figure 3 (an example of data trends extracted from different depth of 3D stacks is shown in Figure S4A,B). The characteristic dimensions for each system, λ, are calculated from eq S1 (Supporting Information file), and listed in Table 1. The average size of blobs increases for all the investigated samples, except for i-PVP_9, where λ remains almost unchanged.

**Table 1:** Values of the Slopes (1/*λ*) of the Curves Shown in Figure 3, the Characteristic Length (λ) for Each System, and the Percentage of λ Increase Due to the FT Process^a^

pre-gel solution	1/λ (slope)	λ (μm)	gel (after FT)	1/λ (slope)	λ (μm)	increase (%)
i-PVA_9	0.59 ± 0.05	1.7 ± 0.1	i-PVA_9	0.49 ± 0.04	2.0 ± 0.2	+20
i-PVP_9	0.40± 0.04	2.5 ± 0.1	i-PVP_9	0.44 ± 0.05	2.3 ± 0.2	–9
i-PVA_12	0.60 ± 0.03	1.7 ± 0.1	i-PVA_12	0.49 ± 0.03	2.0 ± 0.1	+23
i-PVP_12	0.78 ± 0.04	1.3 ± 0.1	i-PVP_12	0.44 ± 0.05	2.3 ± 0.2	+77

a Uncertainties on data were obtained from the fitting procedure.

A comparison among the chord distribution in all pre-gel and gelled systems (Figure S4C,D) shows that blob distribution in *X* = 9 samples is broader (i.e., blobs are more polydisperse) than in *X* = 12. However, the average blob size in gels (λ in Table 1) does not vary significantly in i-PVA or i-PVP series, in agreement with the gel pores observed in Figure 1.

The polymer–polymer phase separation also influences the sub-micron sized pores: the SEM micrographs in Figure S5 show the presence of either very small (PN_5 gel, Figure S5A) or elongated pores (PN_12 gel, Figure S5B) in the PN systems, while the addition of a porogen determines the formation of larger, ellipsoidal pores (Figure S5C–F). As small or needle-shaped pores are not visible in i-PVA or i-PVP samples, we infer that gel morphology, resulting from the water-polymer phase separation occurred during freezing, is completely altered by the presence of a porogen. In this case, the water expelled from the continuous phase during gelation is preferentially collected inside blobs, thus, needle-shaped pores are not observed in sponge-like gels.

Figures 2 and S3 show that for both H-PVA/ L-PVA and H-PVA/ PVP aqueous solutions, phase segregation occurs due to the incompatibility between the two polymer pairs. We recently investigated the phase-behavior of H-PVA/L-PVA (in a 3:1 ratio of the polymers).^18^

H-PVA/ L-PVA mixtures showed a phase separation in concentrated solutions: L-PVA blobs are visible both at high temperatures (100 °C) and room temperature when the total polymer concentration is ≥12% w/v. Polymer–polymer demixing is triggered by lower temperatures for H-PVA/ PVP mixtures (detail of samples of interest are shown in Figure S6).

However, after 1 day-ageing at 25 °C, the solutions showed phase-separation for all the investigated concentrations. PVP forms blobs in the H-PVA continuous phase when the total polymer concentration is ≥12% w/v. The experimental observations and the examination of PVA phase-behavior in the literature (see Supporting Information file for further details) suggest that the observed phase separations are due to two main reasons: (i) L-PVA chains collapsed conformation (argument valid for H-PVA/L-PVA mixtures)^39^ and (ii) tendency of H-PVA chains to self-interact^40^ (argument valid for both polymer mixtures). It follows that L-PVA and PVP chains are expelled from the continuous phase. The collapsed L-PVA conformation in aqueous solution was confirmed through FCS experiments (see further comments on H-PVA/L-PVA and H-PVA/PVP phase behavior, Figure S7 and Table S1 reported in Supporting Information file), while the shear-thinning behavior of pre-gel solutions (Figure S8) supports H-PVA chain self-interactions.

### Physico-Chemical Properties and Structure at the Nanoscale

In pre-gel solutions, higher local concentrations of crystallizable polymer chains (mostly H-PVA chains^18^) facilitate the formation of polymer crystals, which act as tie-points once the gels are formed. Therefore, higher H-PVA concentrations usually lead to gels with a higher crystallinity, i.e., a higher crosslinking degree.^41^

The presence of a second polymer is expected to influence the cryo-structuration process at the nanoscale, as it could alter PVA chains’ ordering and ability to crystallize.

Crystallinity degrees of cryogels are listed in Table 2. Crystallinity of gels after thawing, χ_c__AT, increases with *X* for all the series. The presence of L-PVA or PVP causes a decrease in χ_c__AT. This suggests that L-PVA and PVP (having the largest effect) hinder H-PVA crystallization during the freezing step, particularly at lower H-PVA concentrations.

**Table 2:** Crystallinity Degree after Thawing (χ_c__AT) and Washing (χ_c__AW); Swelling Ratio (*q*_v_); and Gel Fraction (*G* %) of PN, i-PVA, and i-PVP Cryogels^a^

sample	χ_c__AT	χ_c__AW	*q*_v_	*G* %	correlation length (nm)	crystallites radius, *R* (nm)
PN_5	26 ± 1	34 ± 1		27 ± 1	5.7 ± 0.8	3.9 ± 0.2
PN_7	32 ± 2	35 ± 2	1.40	29 ± 1	5.4 ± 0.8	5.0 ± 0.1
PN_9	30 ± 1	35 ± 1	1.77	29 ± 8	5.2 ± 0.8	5.1 ± 0.1
PN_12	33 ± 1	41 ± 5	1.90	30 ± 4	4.9 ± 0.4	5.2 ± 0.1
i-PVA_5	17 ± 2	35 ± 1	2.40	21 ± 1	4.7 ± 0.1	5.3 ± 0.4
i-PVA_7	22 ± 1	36 ± 1	2.15	18 ± 7	4.6 ± 0.1	5.1 ± 0.2
i-PVA_9	27 ± 1	38 ± 1	2.04	27 ± 1	3.4 ± 0.1	6.2 ± 0.2
i-PVA_12	26 ± 1	39 ± 3	2.40	28 ± 1	3.3 ± 0.1	5.9 ± 0.2
i-PVP_5	9 ± 3	19 ± 2	2.51	59 ± 1	3.6 ± 0.1	5.8 ± 0.2
i-PVP_7	11 ± 3	26 ± 6	1.40	38 ± 1	3.7 ± 0.1	5.4 ± 0.1
i-PVP_9	15 ± 2	26 ± 6	1.37	41 ± 2	3.7 ± 0.1	5.2 ± 0.1
i-PVP_12	30 ± 1	33 ± 1	1.48	41 ± 1	4.3 ± 0.1	4.8 ± 0.1

a Averages and standard deviations (3 repetitions at least) are listed. Correlation length and crystallites radius (fitting parameters and errors obtained through the fitting procedure) from the fitting of SAXS curves are listed, as well. Data χ_c__AT for i-PVP series before washing are indicative.

When the thawed gels are swollen and stored in water, non-trapped polymer chains are extracted and a “second crystallization” may occur.^42^ In general, the crystallinity of gels after washing, χ_c__AW, shows the same trend of χ_c__AT. However, the effects of the second crystallization are stronger in both i-PVA and i-PVP series, where the chains of the porogen are preferentially extracted during washing. As a result, H-PVA chains are more likely to interact and form new crystallites. Nonetheless, i-PVP gels are generally less crystalline than their PN or i-PVA counterparts.

The gel fraction (*G %*) indicates active chains of the networks, i.e., those connected through crosslinks. They play a major role in determining gel elasticity and swelling ability (quantified through the equilibrium volume swelling ratio, *q*_v_). *G* % and *q*_v_ of the washed samples are reported in Table 2. *G* % increases with *X* in all the series, as expected. The gel fraction of i-PVP samples is the highest.

The values of *q*_v_ show that L-PVA facilitates gels swelling for all *X* values. PVP, conversely, increases gels elasticity, resulting in lower swelling abilities.

Overall, *G* % and *q*_v_ suggest that i-PVP gels are more rigid (data confirmed by rheology measurements, see Figure S2C and following sections). Nonetheless, their degree of crystallinity, χ_c_, is lower than those of i-PVA and PN networks. We conclude that crystallization is only one of the factors determining the final structure of i-PVP systems.

**Figure 4:**
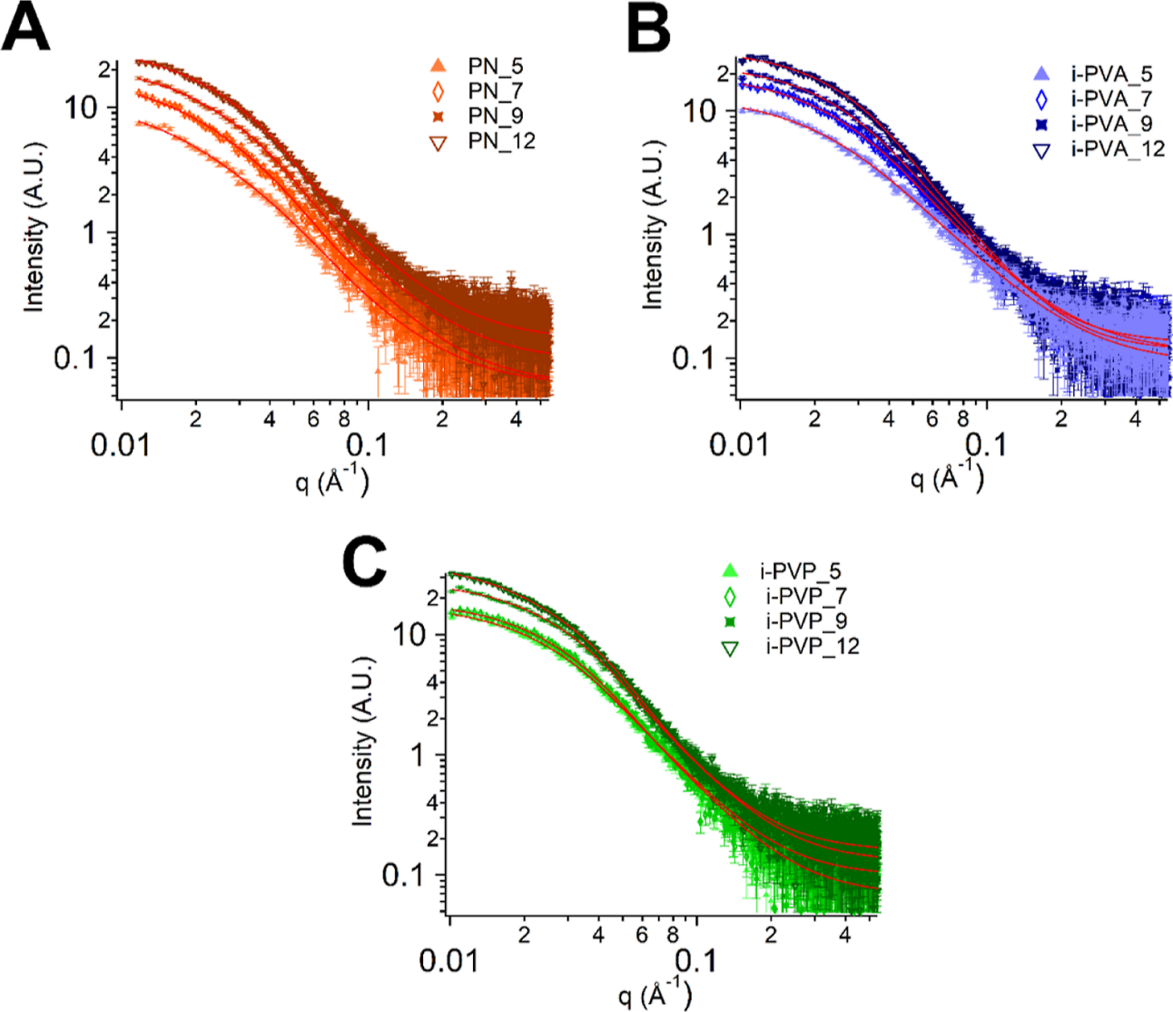
SAXS curves of PN (A), i-PVA (B), and i-PVP (C) gels acquired after thawing.

Further information on gels features at the nanoscale were obtained through SAXS. SAXS curves of the thawed gels (non-washed gels) PN_5-12, i-PVA_5-12, i-PVP_5-12 (Figure 4), were fitted according to eq S4 (Supporting Information file). The complete list of the fitting parameters can be found in Table S2, while the correlation length and the crystallites radii are listed in Table 2. The correlation length, ξ, decreases linearly along the series, suggesting that the gel walls become thicker by increasing H-PVA concentration. ξ values decrease further when L-PVA or PVP are added to the formulation. An abrupt drop in ξ values (from 4.7 to 3.4 nm) occurs in passing from i-PVA_7 to i_PVA_9, as phase-separation occurs in pre-gel solutions (see Figure 2B,C).

The values of crystallites radius, *R*, increase with *X* for PN and i-PVA series, as polymer crystallites are more likely to form at higher H-PVA concentrations. In i-PVA series, the abrupt change between i-PVA_7 and i-PVA_9 is again clearly visible: crystallites radius becomes almost 1 nm larger when H-PVA–L-PVA phase-separation occurs. However, *R* follows for i-PVP series the opposite trend, i.e., it decreases when *X* increases. Considering the unaltered i-PVP_9 blob size before and after freezing, in addition to ξ, *R*, χ_c_, and *G* % trends for i-PVP series, we conclude that the freezing step is partially determining i-PVP systems gelation. H-PVA–PVP phase-separation is complete, meaning that PVP is totally removed during washing (see next section), thus, promoting H-PVA self-interactions already in the pre-gel solution. Therefore, the freezing step only consolidates a system that is already arrested.

In other words, i-PVP gels result from two different gelation steps: a mild physical gelation at room temperature, triggered by H-PVA–PVP phase-separation, followed by cryo-structuration. Therefore, i-PVP gels are less crystalline than their i-PVA counterparts, but more rigid.

### L-PVA and PVP as Structuring Agents

The influence of increasing concentrations of L-PVA or PVP in gels prepared at constant X was investigated. The addition (up to a 3% w/v concentration) of both L-PVA (Figure S9A) and PVP (Figure S9B) leads to more elastic gels (higher storage modulus, *G*′, if compared to PN gel). However, the structuring capacity of L-PVA is lower than PVP. Concentrations higher than 3% w/v lead to a drop in *G*′, both L-PVA and PVP acting as plasticizers.

The point of gelation of H-PVA in i-PVA and i-PVP mixtures, evaluated for 3% w/v L-PVA and PVP concentrations (see Figure S10), further confirms that PVP highly hinders H-PVA crystallization. It also shows that L-PVA actively participates in the final network.

The persistence of L-PVA chains in i-PVA cryogels, after storage in water, was already proven^18^ through confocal microscopy. However, NMR data (see Figure S11A) suggest that the amount of L-PVA permanently embedded in i-PVA networks is almost independent on its initial concentration. On the other hand, washed i-PVP samples (Figure S11B) contain a negligible amount of PVP.

To sum up, i-PVP systems are less crystalline but more rigid than their i-PVA counterparts; moreover, PVP is completely extracted during gels washing, in contrast to L-PVA. Such behavior can be explained considering the high hydrophilicity of PVP, which makes it one of the most common wetting agents, used both in pharmaceutical and cosmetic formulations.^43,44^ In i-PVP pre-gel solutions, PVP blobs subtract water from the H-PVA continuous phase. As a result, H-PVA local concentration and chain entanglement increase. In these conditions, some H-PVA chains interact and form small, unstable crystallites: gelation is already incipient. Once the systems undergo the FT process, the structure is only consolidated: H-PVA chains entanglement prevents an extensive growth of the polymer crystallites, whose size decreases as X increases (see *R* values in Table 2). Besides, the high chain entanglement leads to the formation of stiff networks, characterized by a high *G*′ value.^45^ It is worth noting that PVP does not directly contribute to the gelation process and the polymer is almost completely extracted during washing.

### Gelation Mechanism

The gelation mechanism was investigated through dynamic rheology measurements by observing the dependence of the storage modulus, *G*′, on the concentration of polymer constituting the network.^46^

The gelation process can often be described through the percolation model,^47^ according to which *G*′ is related to the fraction of reacted bonds, *p*, through: , where *p*_c_ is the gelation threshold, i.e., the critical value of *p* to obtain an infinite cluster. For gels well beyond the gelation threshold, swollen in good solvents, the system can be considered a semi-dilute solution of polymer chains. Experimental values of the exponent *n* usually range from 1.9 to 3.5.^48,49^ For PVA cryogels, *n* = 2.4–3.8 have been reported.^50−52^

**Figure 5:**
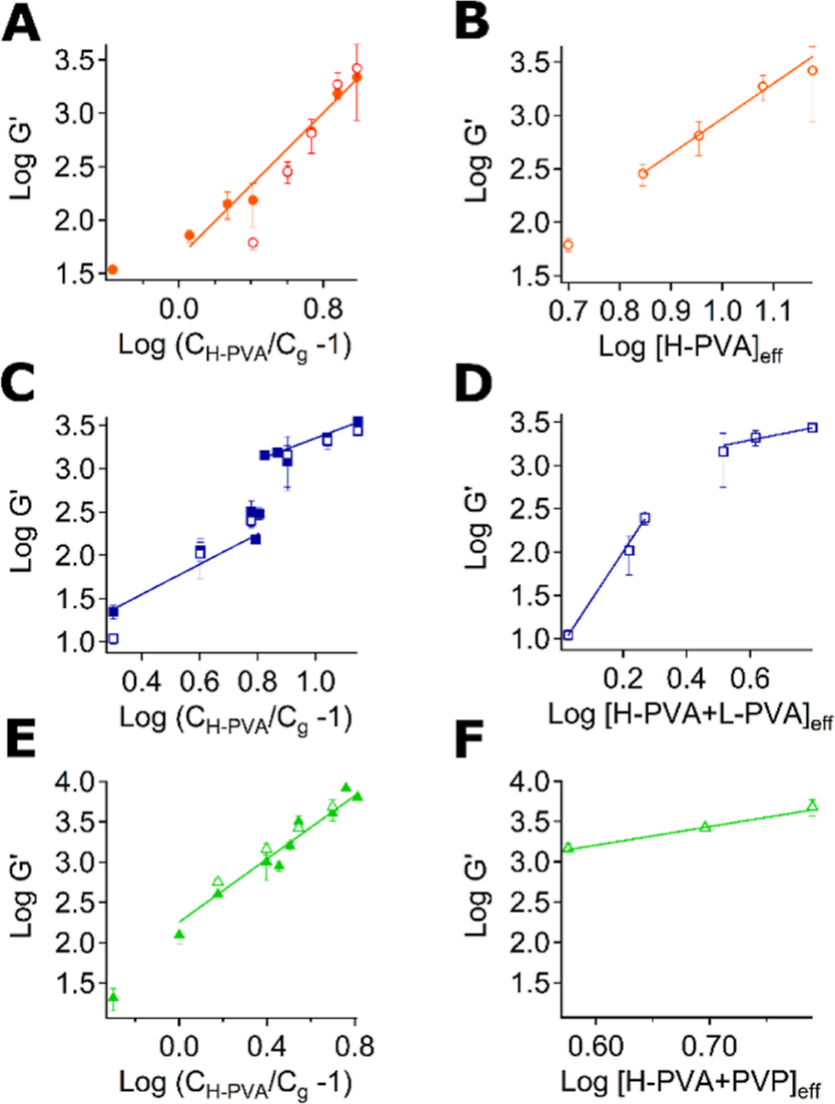
Log–log plots of the storage modulus (*G*′, 1 Hz) vs the effective concentration of polymer of PN, i-PVA, and i-PVP gels. (A,C,E) show the trends of *G*′ vs the effective H-PVA concentration, obtained from the gelation threshold, for PN, i-PVA, and i-PVP gels right after thawing (full markers) and after washing (empty markers). (B,D,F) show the trend of *G*′ of some gel samples of each series after washing vs the effective polymer fraction constituting the networks, calculated using *G* % values. Each data point represents the average of 3–5 frequency sweep measurements, with error bars showing standard deviations.

The rheological behavior of PN, i-PVA, and i-PVP gels was studied after their thawing and washing (Figure 5), to verify the effects related to the extraction of the free polymer chains in water and the second crystallization process. *G*′ data are reported as a function of the effective H-PVA concentration , where *C*_g_ is the gelation threshold (Figure 5A,C,E); the values of the exponent n obtained through the linear fitting are reported in Table S3. The effective polymer concentrations in the washed gels were also calculated, considering the gel fraction, *G* %, of each sample after washing (see Table 2).

In agreement with the loss of polymer chains, in the PN series (Figure 5A) only *G*′ of PN_5 sample changed significantly after washing. Moreover, the fitting of the data for thawed gels supports a gelation process based on a percolation mechanism (see Table S3).

The slope of storage modulus for i-PVP thawed gels (Figure 5D) is also in agreement with a percolation mechanism. On the contrary, the slope calculated in the washed i-PVP series (Figure 5E), describes gels swollen in good solvent, *n* = 2.25,^47^ or, in other words, gels with less structural defects and, therefore, more stable than washed PN.

The *G*′ vs  trend drastically changes when i-PVA networks are considered (Figure 5C). In this case, the curves describing both thawed and washed gels are characterized by two regimes. The presence of two regimes could be the effect of dangling polymer chains and defects in the gel network, which would lead to false values of the exponents at lower polymer concentrations.

Gelation occurring in two different regimes has been already observed^53^ and could be described on the basis of Jones-Marques (JM)^54^ theory on fibrillar gels and Guenet^55^ adaptation to physical gelation processes. Further details on the theories can be found in the Supporting Information file.

Considering JM theory, the presence of the two regimes observed for the i-PVA series (see Figure S12 and Table S3) can be explained considering the presence of dangling polymer chains at lower H-PVA concentrations, which are gradually incorporated in the network as the polymer concentration increases. Assuming that cryogels are made of straight, interconnected fibrils, JM theory allows to extract the fibrils surface fractal dimension, *D*_F_, for different *G′* vs *X* regimes. *D*_F_ = 1 indicates straight elements with a smooth surface, while higher values are related to surface defects. The values of *D*_F_ for i-PVA series are shown in Table S3. Both values are ∼1, indicating the presence of straight elements, while the trend suggests that *D*_F i-PVA_5_ > *D*_F i-PVA_12_. As a matter of fact, *G*′ significantly decreases when i-PVA_5 sample is washed. Thus, structural defects are very likely present in this gel. SEM images (Figure S5) show that i-PVA_5 is characterized by irregular gel strands, with pitched surfaces, that make the whole network defective and, therefore, less stable than i-PVA_12. The latter shows well defined structure, with undamaged strands of different thicknesses.

### Dye Uptake from a Paper Support and Gels Apparent Tortuosity

This work is ultimately aimed at linking the cryogel structural features as pores size and morphology to the gels cleaning capacity of surfaces. The term “cleaning” in the restoration practice refers to the removal of unwanted materials from the artwork surface. In this case, we focused on the removal of hydrophilic dirt, i.e., water-soluble compounds and suspensible particulate matter, such as dust. The cleaning ability of a system depends on several variables: not only the free water and the diffusion of the cleaning fluid through the matrix but also the stickiness could drastically affect the cleaning performances. To simplify the concept of “cleaning”, we decided to consider only the gels transport properties. Specifically, we focused on the diffusion of the water-soluble dye tartrazine through the networks. We evaluated dye uptake from a solid support and gels permeability to a tartrazine aqueous solution. It is worth to mention that tartrazine does not interact with PVA^56^ or with the gels matrix.

**Figure 6:**
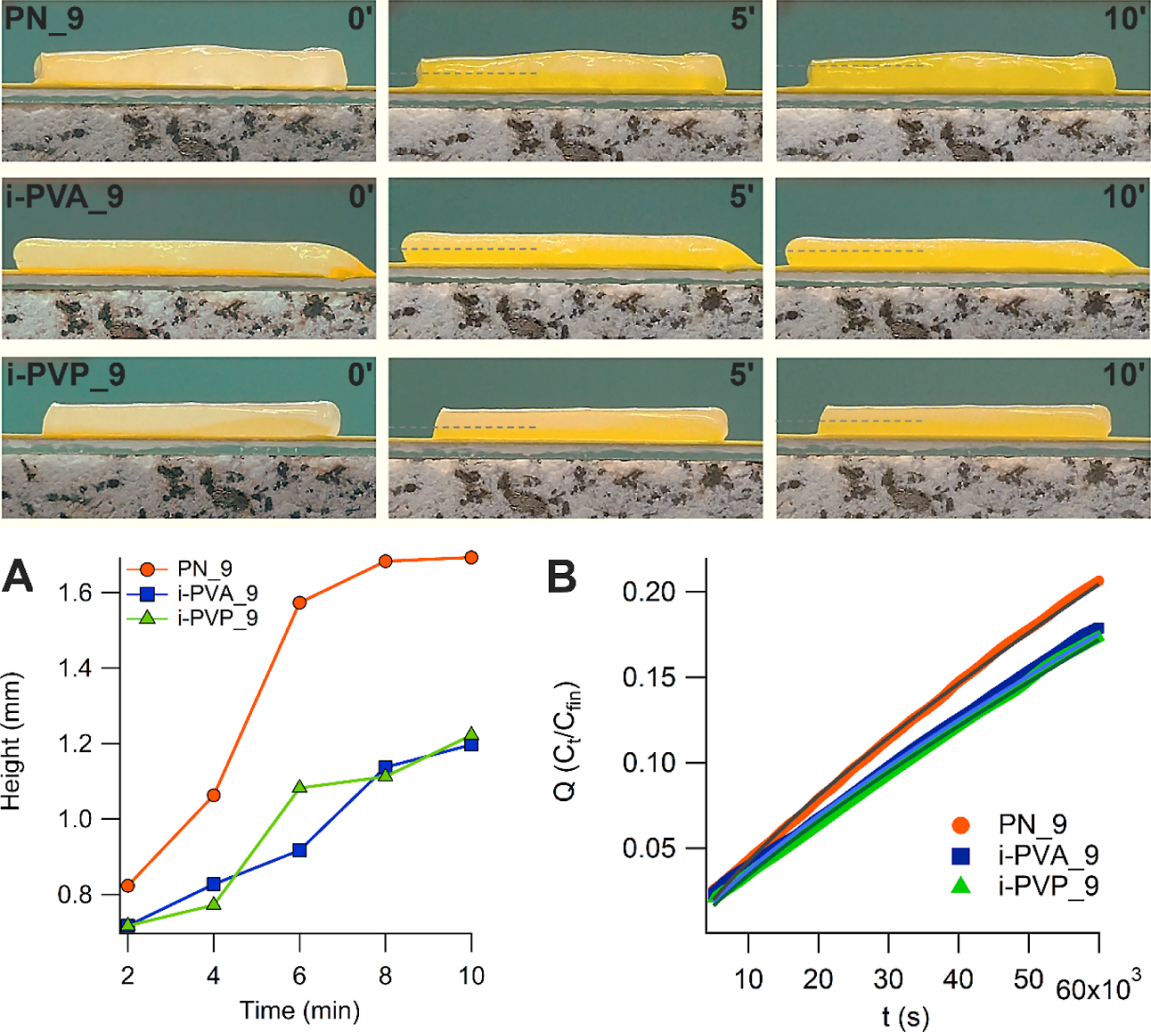
Top panel: gels containing 9% H-PVA (PN_9, i-PVA_9 and i-PVP_9) interacting with a tartrazine-dyed paper sheet. The dye is dissolved at the interface, and the aqueous solution diffuses through the gel network. Height of the dyed solution inside the networks over time: (A) from 0.5 to 10 min; (B) diffusion kinetics of tartrazine, measured in solution through a homemade Franz-diffusion cell.

For uptake experiments, sponge-like systems (*X* = 9, 12) were placed in contact with cardboard sheets, previously soaked in a tartrazine aqueous solution, and then dried (Figure 6, top panels). Gels-paper contact lasted 10 min. The water at the gel-paper interface dissolves the dye, and the dyed solution is subsequently absorbed by the gel matrix. Different processes take place: (i) the concentration gradient recalls water from the upper gel layers to the gel-paper interface; (ii) water evaporation from the gel’s exposed surface recalls water from the gel’s bottom layers; (iii) the dye diffuses through the matrix by Brownian diffusion. All of them contribute to the dye diffusion through the gel sheet.

The gel water release was considered to explain the differences observed among the samples (see Table S6 in Supporting Information). However, a significant difference was found only by comparing *X* = 9 and *X* = 12 gels. In *X* = 9 series, a slight trend emerged (PN 9 > i-PVA_9 > i-PVP_9), in line with the faster dye uptake measured for PN systems. Nonetheless, water release cannot be considered a crucial parameter in the dye uptake process. Even if the factors contributing to the dyed solution uptake are complex, they are related to the hydrodynamic tortuosity factor (obtainable when a gradient of fluid pressure exists across a certain region of a porous material)^57^ that, in turn, depends on the pore size and connectivity.

Figure 6A shows that the rise of the dye solution inside *X* = 9 gels is faster in PN_9 sample. Magnifications showing in more detail the solution front are shown in Figure S13. SAXS data show that the uptake times can be related to the gels correlation lengths. Correlation lengths obtained from the fitting (see Table 2), ξ_PN_9_ > ξ_i-PVP_9_ ∼ ξ_i-PVA_9_, agree with the faster uptakes of PN_9 gel. Similar results have been obtained for *X* = 12 gels, see Figure S14A. Gel permeability and diffusion mechanism were evaluated by using a “homemade” Franz-type diffusion cell, where two chambers (one containing tartrazine aqueous solution, the other milli-Q water) were separated by a gel sheet (see Supporting Information file for further details). Dye diffusion through the gels was evaluated by measuring the tartrazine absorbance increase, in the milli-Q water compartment, over time. The best-fitting of diffusion kinetic curves was obtained by Ritger–Peppas^58,59^ (Figure 6B, eq 3) and Weibull^60^ (Figure S15, eq 4) equations
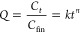
3
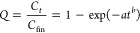
4where *C*_*t*_ is the tartrazine concentration in the second cuvette at time *t*, and *C*_fin_ is the equilibrium concentration of tartrazine. *k*, *n*, *a*, and *b* are constants.

Fitting parameters are listed in Table S4. The exponent *n* in Ritger–Peppas equation is related to the diffusion mechanism: 0.5 < *n* < 1 indicates non-Fickian (anomalous) transport.^58,59^ The parameter *k* is related to gel permeability^60^ and confirms the observations of the dye uptake experiments: the diffusion through PN_9 gel is faster than both i-PVA_9 and i-PVP_9. Regarding Weibull fitting parameters, *b* > 0.8 confirms anomalous transport for all the networks.

**Figure 7:**
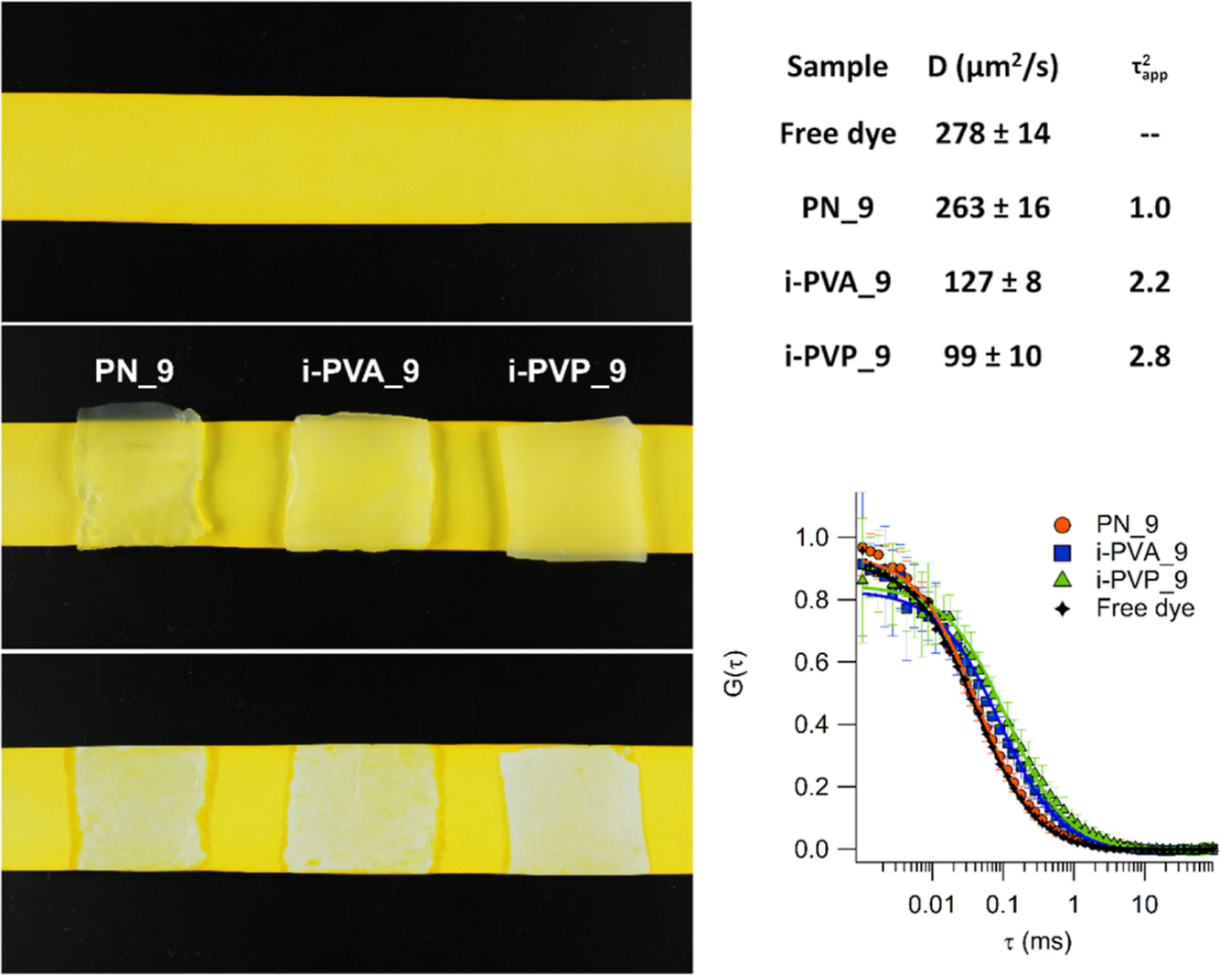
Left panel: cleaning performances of *X* = 9 gels. Top: paper sheet imbibed in a tartrazine solution and dried. Center: PN_9, i-PVA_9, i-PVP_9 during the interaction with the cardboard. Bottom: cleaned areas left after a 10 min contact with the gel sheets; cleaning ability increases with gels apparent tortuosity: PN_9 < i-PVA_9 < i-PVP_9. Right panel, top: diffusion coefficients of Alexa Fluor 568 in the tartrazine solution (free dye), and in gels right after the interaction with the cardboard, obtained from FCS. The *D* values were obtained from the fitting of averaged decays (12–15 repetitions per sample). The uncertainties were obtained from the fitting. The apparent tortuosity factor τ_app_^2^ was calculated as the ratio of *D*_sol_ (diffusion coefficient in tartrazine solution) and *D*_gel_ (diffusion coefficient in gels). Right panel, bottom: FCS curves acquired for the free dye in the tartrazine solution and in gels, after the interaction with the cardboard, and curves fitting.

Gel permeability to water-soluble molecules is expected to be related to gel cleaning ability. Tartrazine removal from dyed cardboards was also evaluated after a 10 min gel–substrate interaction. The treated cardboard are shown in Figure 7 (*X* = 9) and Figure S16 (*X* = 12). The dye removal was quantified on grayscale images, where the average grayscale intensity (listed in Table S6) was calculated in 400 × 400 pixels^2^ squares, centered inside each cleaned area (cleaned areas are about 800 × 760 pixels^2^ large). The results highlight that the total dye uptake increases as PN < i-PVA < i-PVP. Moreover, *X* = 12 gels perform better than *X* = 9.

To relate gel uptake and cleaning abilities to gel tortuosity at the nanoscale, the reciprocal of the gel effective relative diffusivity, *D*_sol_/*D*_gel_ = τ^2^/ε, was calculated.^30,31^ Such parameter includes gels porosity, ε, and their geometrical tortuosity, τ, at the same length scale, that it is directly related to τ^2^, the tortuosity factor. *D*_sol_ and *D*_gel_ represent the diffusion coefficients of an independent dye, Alexa Fluor 568, measured through FCS. Alexa Fluor 568 is a standard fluorescent dye used for FCS calibration.^61^*D*_sol_ represents the diffusion coefficient of Alexa Fluor 568 in a concentrated Tartrazine aqueous solution. To obtain *D*_gel_, gels imbibed with a dilute solution of Alexa Fluor 568 were placed in contact with the Tartrazine-soaked paper support for 10 min. The Alexa Fluor diffusion coefficient through the gel matrix was measured immediately after the interaction. FCS curves are shown in Figures 7 and S14B, for *X* = 9 and *X* = 12 gels, respectively.

The ratio between the tortuosity and gels porosity, τ^2^/ε, (apparent tortuosity ) was calculated for *X* = 9 and *X* = 12 gels. The obtained values are listed in top right panel of Figure 7 and Table S5. These data show that  is influenced by the presence of tartrazine, meaning that the  is not an independent parameter. Nonetheless, as previously stated, tartrazine does not interact with PVA^56^ or the gel matrix. Therefore,  is related to Alexa Fluor molecules effective path length,^50^ i.e., to the nanoscale tortuosity, and provides detailed information of each gel cleaning mechanism. It was found that  of PN samples is significantly lower in the case of both *X* = 9 and *X* = 12 series, suggesting that PN systems nano-scale pores are probably elongated and “straight-lined,” as those observed at the micron-scale. This further supports the dependence of the pore morphology, at all lengthscales, on the presence of a polymer–polymer phase separation: as already observed through confocal and SEM imaging, pores shape and size change drastically when L-PVA or PVP are added to the formulations.

Considering that the dye uptake is faster and  lower for the PN systems, we expected that the PN samples had the best cleaning performance. However, the experimental results showed the opposite. These results are contradictory only in the appearance. The residence time of the aqueous solution at the cardboard-gel interface is a crucial factor to consider for the cleaning process. In PN gels the uptake is so fast that only lower amounts of tartrazine can be removed from the gel-cardboard contact area, before the interface is repopulated by tartrazine molecule from the inner section of the dry cardboard. Sponge-like gels, on the other hand, grant longer residence times of the “cleaning medium” at the interface: this allows higher tartrazine dissolution, and higher final uptakes. PVA-based sponge-like gels have been already proven the best cleaning tools in the removal of water soluble and suspensible dirt from masterpieces by Jackson Pollock.^19^ These results are extremely important, showing that the cleaning capacity and kinetics can be controlled and tuned by changing the gels transport properties and  parameter. However, further insights into gels transport properties and tortuosity at different length scales are still needed to achieve a complete and detailed understanding of the cleaning process.

## Conclusions

Cryogels with tailored pore size were obtained exploiting the phase-behavior of H-PVA–L-PVA (i-PVA) and H-PVA–PVP (i-PVP) mixtures. Their structural properties were compared to those of neat H-PVA (PN) gels. Confocal micrographs revealed that the gels pores morphology and size are affected by a polymer–polymer demixing, occurring in the pre-gel solutions. When demixing occurs, for both i-PVA and i-PVP mixtures, L-PVA and PVP concentrate in blobs and act as porogens during cryostructuration. Blobs size generally increases after the FT process, leading to sponge-like gels after thawing. It is worth noting that the polymer–polymer phase-separation is the major contributor determining pores morphology in i-PVA and i-PVP systems, overruling the needle-shaped porosity formed when homogeneous systems, like PN pre-gel solutions, undergo a FT process. The addition of L-PVA or PVP affects H-PVA crosslinking ability, leading to gels with lower crystallinity right after the FT process. During phase separation, H-PVA concentrates in the continuous phase causing an increase of H-PVA crystallite size in i-PVA systems. For i-PVP gels, crystallite size is independent on H-PVA concentration: H-PVA–PVP phase separation in pre-gel solution causes a mild H-PVA physical gelation already before freezing. More specifically, PVP subtracts water from the continuous phase, leading to higher H-PVA chains entanglements and self-interactions. As a result, small H-PVA crystallites would already form in i-PVP concentrated pre-gel solutions. The freezing step consolidates such structures, resulting in stiffer networks. L-PVA alters the gelation mechanism: while PN and i-PVP gels form according to a percolation process, i-PVA gels show two different gelation regimes and can possibly be described as fibrillar networks, according to the Jones-Marques theory. Overall, L-PVA and PVP affect the gels structure, porosity, and physico-chemical properties, acting both as porogens and “structuring” agents. Even if the complete understanding of the cleaning process requires much more effort and the investigation of the interplay between several factors (e.g., uptake of solid particles, gel surface adhesivity, and roughness), we were able to relate the pores size and connectivity to the gels cleaning ability. The uptake of a water-soluble dye from a flat substrate (i.e., paper cardboard), and the dye diffusion across the gels, showed, as expected, that the higher the gel apparent tortuosity, the slower the dye diffusion across the gel. However, we found that higher apparent tortuosity is related to a higher dye removal: this seems contradictory, as longer migration paths for the dye are expected to be related to worse cleaning abilities. Rather, the residence time of the cleaning solution at the gel/substrate interface is a crucial factor to be considered. Longer residence time at the interface, i.e., higher networks tortuosity, allow higher soluble components removal. Thus, the accurate design of gel pores architecture is the cornerstone to obtain efficient removal, achieving optimal cleaning performances.

These results pave the way to controlled targeted applications in a variety of fields: restoration, where sponge-like gels have shown ideal cleaning performances, filtration, with exclusion of particulate and pathogens, cell growth, adhesion and entrapment, wound dressing and tissue engineering, or sorbents.

## Supporting Information Available

Further details on materials and methods; fitting equations (SAXS, FCS, diffusivity); further comments on H-PVA–L-PVA and H-PVA–PVP phase-behavior; further comments on SAXS fitting results; further comments on Jones-Marques (JM) theory and its application to physical gelation; 3D confocal images of PN, i-PVA and i-PVP systems; frequency sweeps of PN, i-PVA, and i-PVP systems; confocal images of i-PVA and i-PVP gels containing RBITC-labeled L-PVA and PVP; chord length distribution of X = 9 and X = 12 systems; SEM micrographs; phase behavior of i-PVP solutions; FCS curves of the three labeled polymers in diluted aqueous solution; shear thinning behavior; log-log plots of the storage modulus; gel points for PN, i-PVA and i-PVP series; NMR spectra of i(Y)-PVA and i(Y)-PVP gels; log-log plots of the storage modulus (i-PVA systems, JM theory); magnifications of highly contrasted pictures showing the dye uptake; experiments of dye uptake and dye diffusion; Weibull fitting (solid lines) of kinetic curves; cleaning performances of X = 12 gels; diffusion coefficients and hydrodynamic radii; complete SAXS fitting parameters; values of the exponent n obtained for the gels after thawing; fitting parameters of diffusion kinetic curves; diffusion coefficients; and water release and dye removal of gels (PDF)
