# Supplementary material for: Sponge-like Cryogels from Liquid–Liquid Phase Separation: Structure, Porosity, and Diffusional Gel Properties
Source: ACS Appl Mater Interfaces. 2023 Jul 29;15(39):46428–39. doi: 10.1021/acsami.3c03239 (PMC10561144; doi:10.1021/acsami.3c03239)
Supplement: Supplementary file 1 — am3c03239_si_001.pdf [file am3c03239_si_001.pdf]

Supporting information for

Sponge-like cryogels from liquid-liquid phase separation: structure, porosity and diffusional gel properties

Rosangela Mastrangelo, Claudio Resta, Emiliano Carretti, Emiliano Fratini and Piero Baglioni**

Department of Chemistry and CSGI, University of Florence, via della Lastruccia, 3, Sesto Fiorentino, Florence, 50019, Italy

Corresponding authors Email: baglioni@csgi.unifi.it, rosangela.mastrangelo@unifi.it

MATERIALS AND METHODS

Cryogel preparation. To sum up, cryogels series are coded as follows:

- PN_X, with X = 3, 5, 7, 9, 12, 15 % w/v, are cryogels containing increasing concentrations of H-PVA (X);
- i-PVA_X, with X = 3, 5, 7, 9, 12, 15 % w/v, are cryogels containing 3% w/v of L-PVA and increasing concentrations of H-PVA (X);
- i-PVP_X, with X = 3, 5, 7, 9, 12, 15 % w/v, are cryogels containing 3% w/v of PVP and increasing concentrations of H-PVA (X);

To investigate the effect of increasing amounts of porogen polymers on a 9% w/v H-PVA network, the following samples were prepared (see Rheology and NMR experiments):

- i(Y)-PVA, with Y = 0.5, 1, 2, 3, 4, 5 % w/v, are cryogels containing 9% w/v of H-PVA and increasing concentrations of L-PVA (Y);
- i(Y)-PVP, with Y = 0.5, 1, 2, 3, 4, 5 % w/v, are cryogels containing 9% w/v of H-PVA and increasing concentrations of PVP (Y);

The same name, when specified, was used also to describe the pre-gel solution.

All measurements were performed on gels right after thawing, and after washing. Washing consists in storing gel sheets in ca. 1.5 L of demineralized water, to extract the soluble components. Water was changed daily (for 7 days).

PVP labeling. PVP was labeled with RBITC, following the same procedure used for L-PVA. In this case, the unavoidable presence of -NH_2 or -OH bearing defects on the backbone was exploited. RBITC labeled PVP was purified by means of several dissolution/precipitation cycles (in water and ethanol) and subsequent dialysis against water. The effectiveness of covalent labeling was proved by fluorescence correlation spectroscopy experiments on labeled PVP water solutions, which showed a diffusion coefficient consistent with a chemically bound probe.

Confocal Laser Scanning Microscopy (CLSM) Imaging. CLSM experiments were performed on a Leica TCS SP8 confocal microscope (Leica Microsystems GmbH, Wetzlar, Germany), equipped

with a 63X water immersion objective. In particular, 3D images (stack of 150 – 170 2D images) were acquired of pre-gel and gelled systems. Images were collected in different spots of the samples, and reproducibility was checked for different gel batches. Pre-gel solutions measurements were performed on samples 24 hours after preparation, while gel samples imaging was performed on washed gels (7 days washing/storage in demineralized water).

3D Image analysis. 3D confocal images of i-PVA_9, i-PVA_12, i-PVP_9 and i-PVP_12 (i.e. all samples containing blobs) were analyzed through the chord analysis distribution[1] method. A characteristic dimension of blobs, λ, was extracted. To implement chord analysis, the MATLAB® algorithm developed by M. Ryan MacIver[2] was modified to work on the Leica format (.lif), i.e. the format of 3D stacks obtained directly from the microscope software. 100 2D images of each confocal stack (thickness of 50 μm ca.) were binarized, and a set of 10,000 randomly oriented lines was drawn on them. Chords are the segments obtained when a line crosses phase-boundaries. . Chords of certain lengths are binned according to their occurrence generating a the plot of the frequency, *f(R)*, *versus* their dimension, *R* (μm). The trends shown in Fig. 3 are averages of the original data points. The slope of the exponential decay in the semi-log graph can be used to obtain a characteristic length, λ_{Blobs}:

$$f_{Blobs}(R) \propto \exp\left(-\frac{R}{\lambda_{Blobs}}\right) \qquad \text{(S1)}$$

In Eq. 1, 1/λ represents the slope of the function in the semi-log graph, while λ is called persistence length. The minimum chord length was set to 2 pixels. Uncertainties on data were obtained from the fitting procedure, implemented through the software Igor Pro 6.37.

Small-angle X-ray scattering (SAXS). SAXS curves were collected with a HECUS S3-MICRO SWAXS-camera (Hecus XRS, Graz, Austria), equipped with a Kratky collimation system and a position-sensitive detector (PSD 50M,), with 1024 channels (width = 54 μm). A Cu anode provided Cu Kα radiation (i.e., 0.1542 nm), by using a 50 W microfocus X-ray tube powered by a GeniX

generator (Xenocs, Grenoble, France) and customized with a FOX-3D single-bounce multilayer point focusing optics (Xenocs) to cut the Cu Kβ emission. The sample-to-detector distance was 281 mm as calibrated by silver behenate (d = 58.38 Å)[3]. The volume between the sample and the detector was kept under vacuum during the measurements to minimize scattering from the air. Scattering curves were obtained in the scattering vector, q, range between 0.009-0.54 $Å^{-1}$. Temperature was kept at 25 ± 0.1°C by a Peltier element. Thawed gel samples (non-washed gels) were cut in thin slices and put in the appropriate sample holder, i.e. a demountable sealed cell for solids, with Kapton as a window material (optical path = 1 mm). Standard measurement conditions were 50 kV, 1 mA. The acquisition time was from 1 to 2 hours depending on the sample. Scattering curves were corrected for the empty cell/water contribution.

The fitting of the SAXS curves was implemented through the software SASView[4]. The scattered intensity, I(q), was modelized as follows[5,6]:

$$I(q) = I_L(0)\frac{1}{\left[1+\frac{D+1}{3}(q^2\xi^2)\right]^{D/2}} + I_G(0)exp\left(-q^2R^2/3\right) + Bkg \qquad \text{(S2)}$$

The two terms describe the scattering due to polymer chains in a liquid-like environment, and the scattering of solid-like objects (physical polymer junctions), respectively.

$I_L(0)$ and $I_G(0)$ are called Lorentzian and Guinier parameters, and define the relative weight of the two terms in the equation; D is the fractal exponent, ξ is the correlation length of non-crystalline polymer chains and R is the radius of gyration of solid-like objects (polymer crystallites).

Fluorescence Correlation Spectroscopy (FCS). A Leica TCS SP8 confocal microscope (Leica Microsystems GmbH, Wetzlar, Germany) equipped with a PicoQuant FCS modulus (PicoQuant, Berlin, Germany) was used to perform FCS measurements. A 63X/1.2 W water immersion objective (Zeiss) was used. The diffusion of FITC-labeled H-PVA in dilute solution ([FITC] = 50 nM) was measured by exciting FITC with the Ar laser line (488 nm) and collecting the fluorescence signal with a Hybrid SMD detector (498-540 nm). The diffusion of RBITC-labeled L-PVA and PVP in

dilute solution ([RBITC] = 50 nM), instead, was measured by exciting RBITC with the DPSS 561 laser line (561 nm) and collecting the fluorescence signal with a Hybrid SMD detector (571–630 nm). Aqueous solutions of Alexa Fluor 568 (25 nM) and Rhodamine 110 (10 nM) were used for FCS calibration[7]. The fitting model assumes that the three-dimensional Brownian diffusion of labeled molecules across a 3D-ellipsoidal Gaussian volume is the only contribution to the observed decay time. FCS curves were averaged (12 – 15 repetitions) and analyzed considering either a one-component decay:

$$G(\tau) = \frac{1}{N}\left[\left(1+\frac{\tau}{\tau_D}\right)^{-1}\left(1+\frac{\tau}{S^2\tau_D}\right)^{-1/2}\right] \tag{S3}$$

or a two-components decay:

$$G(\tau) = \frac{1}{N}\left[f_1\left(1+\frac{\tau}{\tau_{D1}}\right)^{-1}\left(1+\frac{\tau}{S^2\tau_{D1}}\right)^{-\frac{1}{2}} + (1-f_1)\left(1+\frac{\tau}{\tau_{D2}}\right)^{-1}\left(1+\frac{\tau}{S^2\tau_{D2}}\right)^{-\frac{1}{2}}\right] \tag{S4}$$

where N is the average number of fluorescent molecules detected inside the confocal volume ($N = CV$, with $V = \pi^{3/2}{w_0}^3 S$ and C the concentration), f_1 is the percentage of the contribution of $\tau_{D,1}$ to the total decay time, $\tau_{D,i}$ are the decay times, and $S = z_0/w_0$ is the ratio between the axial and the lateral dimensions of the confocal volume, determined through the calibration procedure with Alexa 568. The diffusion coefficients D_i of the labeled species can be determined by:

$$\tau_{D,i} = \frac{{w_0}^2}{4D_i} \tag{S5}$$

The hydrodynamic radii of labeled H-PVA, L-PVA and PVP were calculated trough the Stokes-Einstein equation.

The diffusion of Alexa Fluor 568 inside the gels was measured after dye-uptake experiments from tartrazine-dyed cardboards (10 minutes contact) to determine the effective relative diffusivity in gels More specifically, the diffusion coefficient of Alexa Fluor 568 was calculated in a concentrated tartrazine solution (2.5% w/w), the same used to dye the cardboards, and for gels, after tartrazine loading. Details on the effective relative diffusivity calculation are reported in the relative section.

Rheology. Rheology measurements were performed by using a Discovery HR-3 rheometer from TA Instruments (40 mm diameter parallel plate geometry), equipped with a Peltier temperature control system. Amplitude and frequency sweeps were performed on 40 mm diameter gel disks (thickness of 2 mm ca.), while flow curves were acquired on pre-gel solutions, at constant temperature (25°C). The Linear Viscoelastic Range (LVR) was identified through amplitude sweep tests, performed at 1 Hz of frequency, with oscillation strain ranging between 0.01 and 50%. LVR lies between strains of 0.01-10 % for the cryogels. Frequency sweeps curves were thus recorded within this range, at constant oscillation strain (1 %), by increasing the oscillation frequency (0.1-100 Hz). Data of the storage moduli, G', at 1 Hz, are plotted against the effective polymer concentration in the network (obtained either by the fraction of "reacted bonds" of percolation theory, or by G% data) in the *Gelation Mechanism* Section. G' values are averages of three-five measurements.

Nuclear Magnetic Resonance (NMR). NMR experiments were performed on a Bruker Avance Spectrometer operating at the frequency of 400 MHz for ^{1}H in DMSO-d_6. The signal due to residual proton of the solvent was used as internal reference. The amount of porogen polymer after washing and storage in water (2 months) was calculated for i(2)-PVA, i(3)-PVA, i(4)-PVA and i(5)-PVA samples by comparing the signals due to residual esters moieties of L-PVA with alcohols' –CH- of L-PVA and H-PVA, according to the following equation:

$$\frac{L-PVA\,(wt)}{H-PVA\,(wt)}=\frac{\left(\frac{I_{1.9-2.1}}{3\times(1-HD_{L-PVA})}\times MW_{L-PVA}\right)}{\left[\left(\frac{I_{1-1.7}}{2}-\frac{I_{1.9-2.1}}{3\times(1-HD_{L-PVA})}\right)\times MW_{H-PVA}\right]} \quad \text{(S6)}$$

where $I_{1.9\text{-}2.1}$ and $I_{1\text{-}1.7}$ indicate the peaks areas due to the 3 acetate's hydrogens (present in L-PVA only) and the 2 vinyl hydrogens (present in both H-PVA and L-PVA), respectively; HD is the hydrolysis degree of L-PVA; $MW_{L\text{-}PVA}$ $MW_{H\text{-}PVA}$ are the average molecular weights of H-PVA and L-PVA monomers, respectively. Assessment of residual PVP in i(2)-PVP, i(3)-PVP, i(4)-PVP and i(5)-PVP gels was attempted following a similar procedure and, though a trend can be identified, exact quantification was not possible because of its very low amount (less than 6 wt% with respect to H-PVA).

Effective relative diffusivity calculation and relationship with tortuosity. The reciprocal of the effective relative diffusivity[8,9] in gels, ${\tau^2}_{app} = \tau^2/\varepsilon$, was calculated as follows:

$$ {\tau^2}_{app} = \frac{\tau^2}{\varepsilon} = \frac{D_{sol}}{D_{gel}} \qquad \text{(S7)} $$

Here τ^2 is the tortuosity factor, i.e. the square of geometrical tortuosity, and ε is the networks porosity. τ^2/ε was evaluated for tartrazine-loaded gels, *i.e.* after the interaction with tartrazine-dyed cardboards. Thus, the reciprocal of the effective relative diffusivity is directly related to gels apparent tortuosity factor, as it is obtained for gels containing a tartrazine aqueous solution. D_{Sol} and D_{Gel} are the diffusion coefficients of the non-interacting dye Alexa Fluor 568 in tartrazine solution and inside the gels, respectively. Further information can be found in the description of FCS measurements.

Further comments on H-PVA – L-PVA and H-PVA – PVP phase behavior

PVAs with hydrolysis degree (HD) > 80 % are known to be water-soluble polymers[10,11]. When we investigated the phase behavior of solutions containing both H-PVA and L-PVA[12], we found incompatibility for concentrations of polymers (H-PVA/L-PVA ratio of 3:1) equal or higher than 12% w/w, in the range from 5 to 100 °C: spherical blobs of L-PVA formed in the continuous H-PVA phase. Blobs were detectable also for concentrations of 9 and 7 % w/w, but only above 60°C and 70°C.

Polymer-polymer incompatibility can be influenced by the conformation of polymer chains in solution. Lewandowska *et al.*[13] reported about the thermodynamic and hydrodynamic interactions among polymer chains of PVA with higher (98-99%) and lower (85-87%) HD in aqueous solution. They demonstrated that, even though water is a good solvent for both polymers, PVA with high HD tends to self-associate in aqueous solution. Thus, concentrated solutions of highly hydrolyzed PVAs are not stable: they form physical networks, because of the tendency of this type of PVA to crystallize. On the other hand, aqueous solutions of partially hydrolyzed PVAs are more stable, due to the presence of acetates and the subsequent lower tendency to self-associate. The hydrodynamic radii of the labeled H-PVA and L-PVA, in dilute solutions, were calculated from FCS data (fig. S4 and Table S1): H-PVA radius is slightly larger, being 12 nm ca., while L-PVA radius is 9 nm ca. L-PVA radius is low, if compared to PVA with the same HD and similar molecular weight.[14] In light of the previous arguments, H-PVA – L-PVA segregation could be due to L-PVA collapsed conformation and to H-PVA preferential self-interaction.

The inter- and intra-molecular H-bonds[10] forming between H-PVA chains result in a pronounced shear thinning behavior, which is enhanced when the solutions are subjected to steady shear[15] or when the polymer concentration increases[16]. Both i-PVA and i-PVP concentrated pre-gel solutions showed shear-thinning behavior (see fig. S5), that increases with H-PVA concentration.

PVP is a water-soluble polymer, as well. H-PVA – PVP interactions should be favored (negative values of $\chi_{PVP-PVA}$ are reported in the literature[17,18]). In fact, PVA – PVP blends are usually

described as homogeneous mixtures with a single glass transition temperature, and the two polymers are considered as compatible.[19–24] However, their mutual miscibility decreases as their M_w, or the HD of PVA[19], increase.

PVP chains, as opposed to those of L-PVA, are not collapsed in aqueous solution.[25,26] The hydrodynamic radius of a single PVP chain at room temperature, obtained by FCS data (fig. S4 and Table S1), is 25 nm ca.

Further comments on SAXS fitting results

While the gyration radius, *R*, gives insights about PVA crystallites dimensions, the $I_G(0)/I_L(0)$ ratio provides further proofs on the abundance of crystalline portions in the samples if compared to the network portion. The ratio (Table S2) confirms that the number of crystallites increases with H-PVA concentration in PN and i-PVA gels, while, in i-PVP series, a clear trend cannot be identified.

The fractal dimension, *D*, of PN_5-12 and i-PVA_5,7 is similar to the fractal dimension of a polymer solution in semi-dilute regime. In fact, when *D* is about 2, the first term of the fitting equation takes the form of the Ornstein-Zernicke equation. Higher *D* values (2.7 – 2.9), instead, characterize systems with a significant number of H-bonds[27] This is the case of i-PVA_9 and i-PVA_12 samples.

Further comments on Jones-Marques (JM) theory and its application to physical gelation

Gelation occurring in two different regimes, as that of i-PVA series, has been observed for agarose gels[28], and can be justified on the basis of the Jones-Marques (JM) theory. Guenet[29] adapted this model to the physical gelation processes characterizing fibrillar gels. He described fibrillar gels as thermoreversible networks, obtained by the connection of straight fibrils (cross-section of 2-20 nm), with micron-sized pores. PVA chains do not exactly form fibrils in solution, that associate to build a network. However, a PVA cryogel can be described as an array of rigid objects connecting at junctions, due to the high directionality given by both the self-association of H-PVA chains in solution and the freezing step; in fact, cryogels have already been defined fibrillar networks.[30–32] Clearly, the morphology of these gels is complex and the fibrillar description is not exhaustive, but it can be used as a starting point to shed light on the gels formation mechanism.

More specifically, in fibrillar gels, a transition from enthalpic elasticity to entropic elasticity occurs by increasing the polymer concentration. Enthalpic elasticity is linked to rigid tie-points, while entropic elasticity implies the formation of “freely-hinged” junctions, i.e., crosslinks with a certain rotational freedom. According to JM theory, the exponent n can be related to a longitudinal fractal dimension (D_F), i.e. the fractal dimension of objects at junctions: $n = (3 + D_F) / (3 - D_F)$ for enthalpic elasticity, while $n = 3 / (3 - D_F)$ for entropic networks. Therefore, D_F can be used to quantify the fibrils defects: when its value is closer to 1, fibrils are less defected; conversely, for $D_F > 1$, structure perfectioning occurred and fibrils are smooth and homogeneous.

Additional Figures.

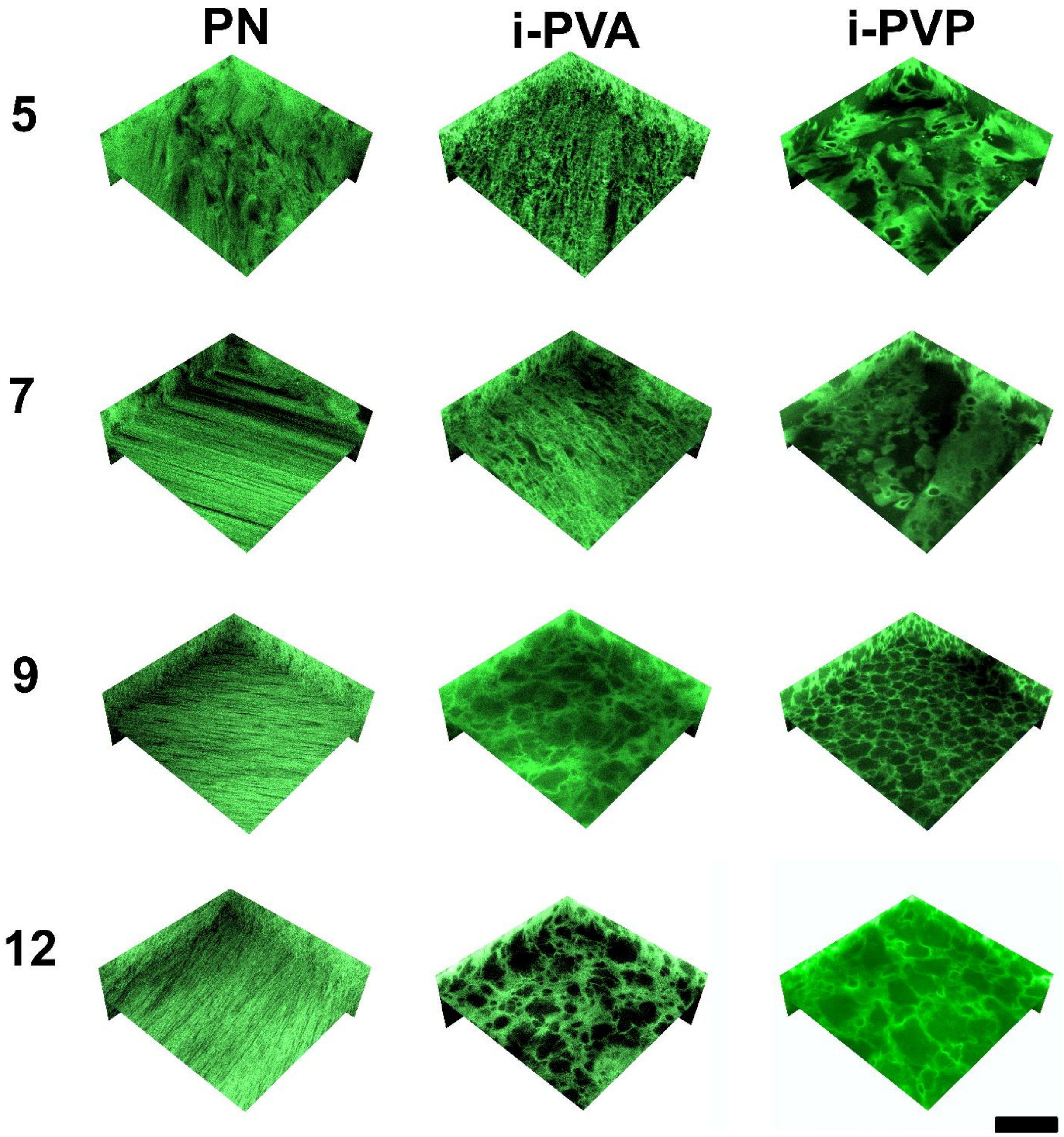

Figure S1. 3D confocal images of PN, i-PVA and i-PVP systems. 2D images shown in Fig.1 were extracted from stacks of this type. Scalebar: 50 µm.

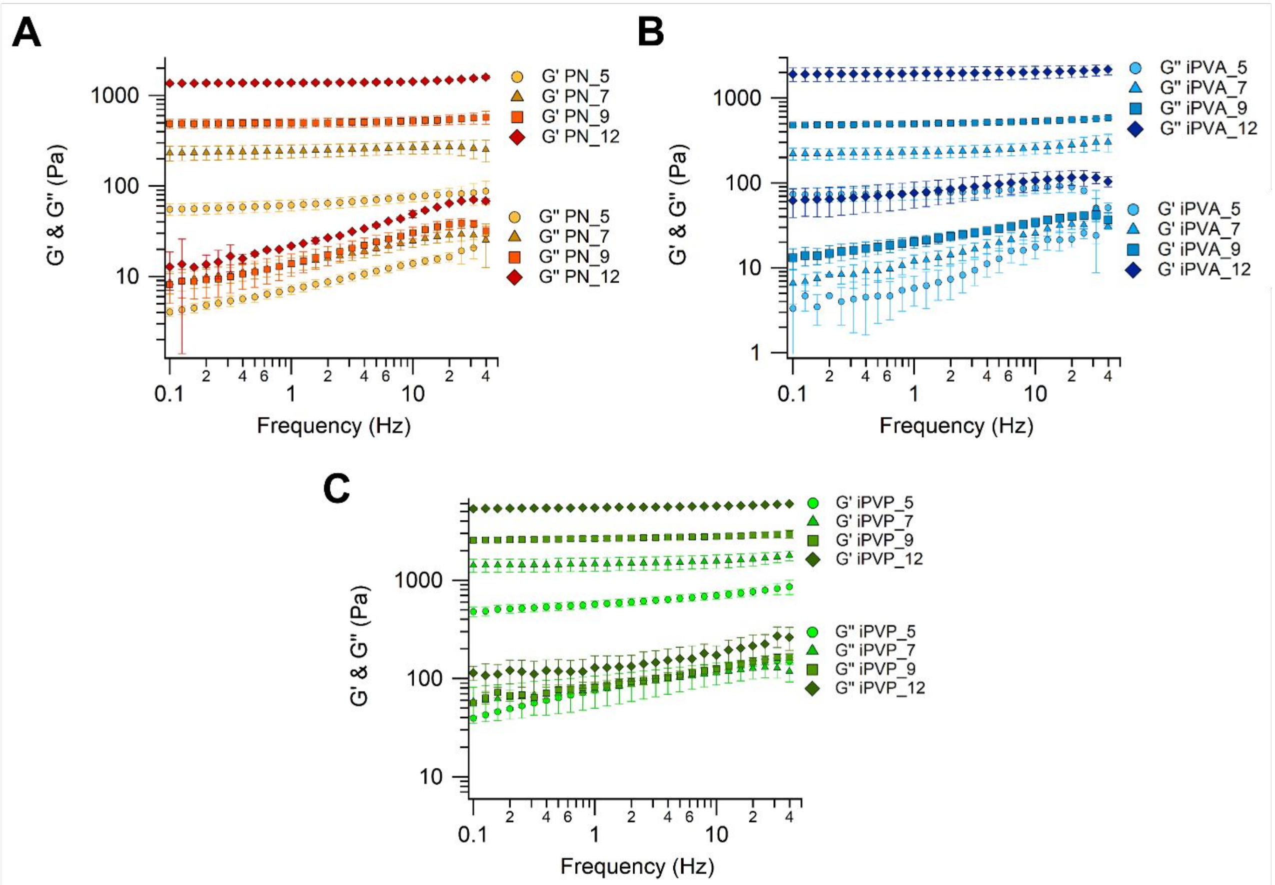

Figure S2. Frequency sweeps of A) PN, B) i-PVA and C) i-PVP systems.

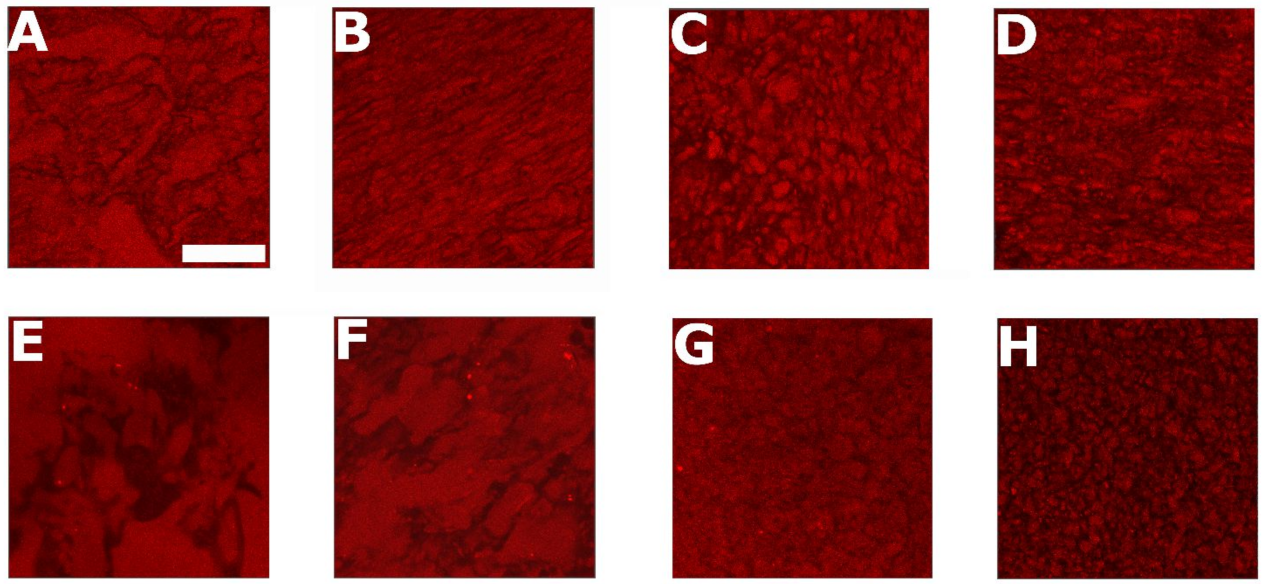

Figure S3. Confocal images of i-PVA and i-PVP gels containing RBITC-labeled L-PVA and PVP: i-PVA_5 (A), i-PVA_7 (B), i-PVA_9 (C), i-PVA_12 (D), i-PVP_5 (E), i-PVA_7 (F), i-PVA_9 (G), i-PVA_12 (H). Scalebar: 50 µm.

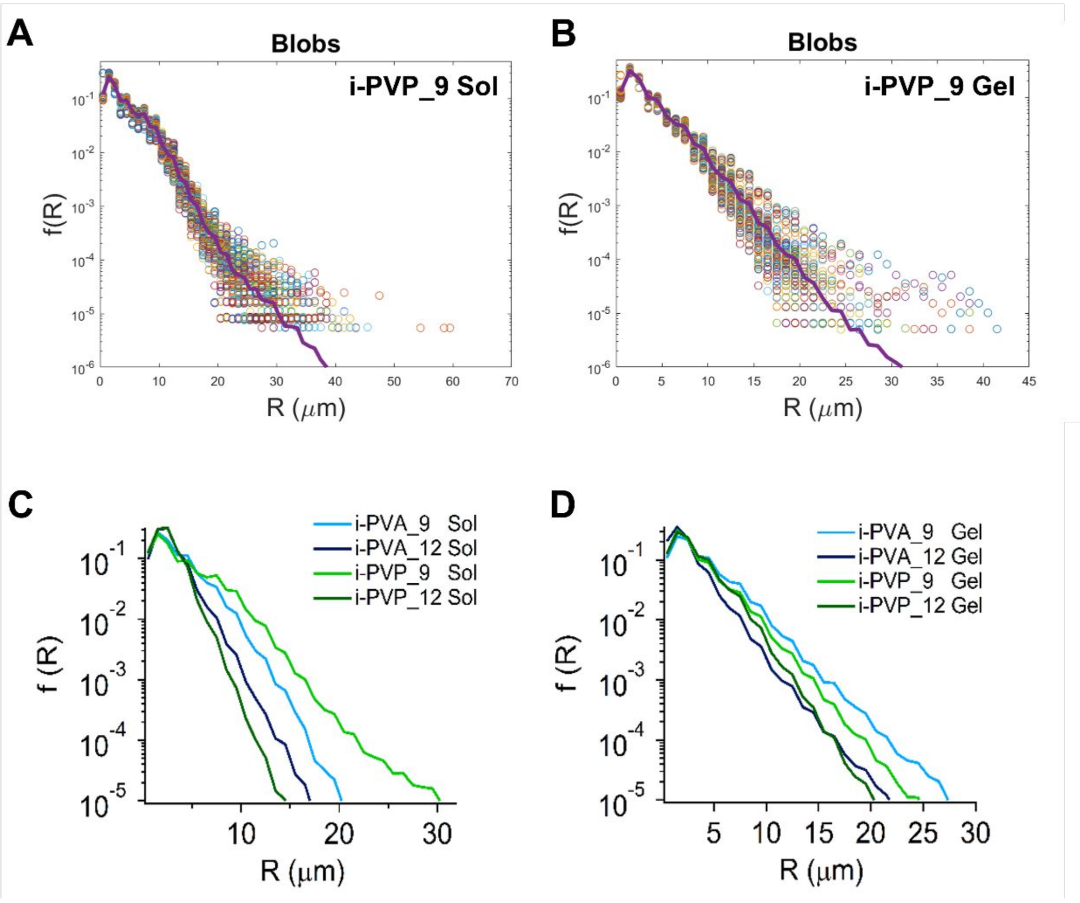

Figure S4. Chord length distribution of X = 9 and X = 12 systems. A-B) Example of data elaboration: different curves (data points f the same color) were exctracted from different 2D images composing 3D confocal stacks, and averaged to give the purple line; A) i-PVP_9 Sol; B) i-PVP_9 gel; C – D) Averaged curves obtained for i-PVA_9, i-PVA_12, i-PVP_9 and i-PVP_12 pre-gel solutions (C) and gels right after the FT process (D).

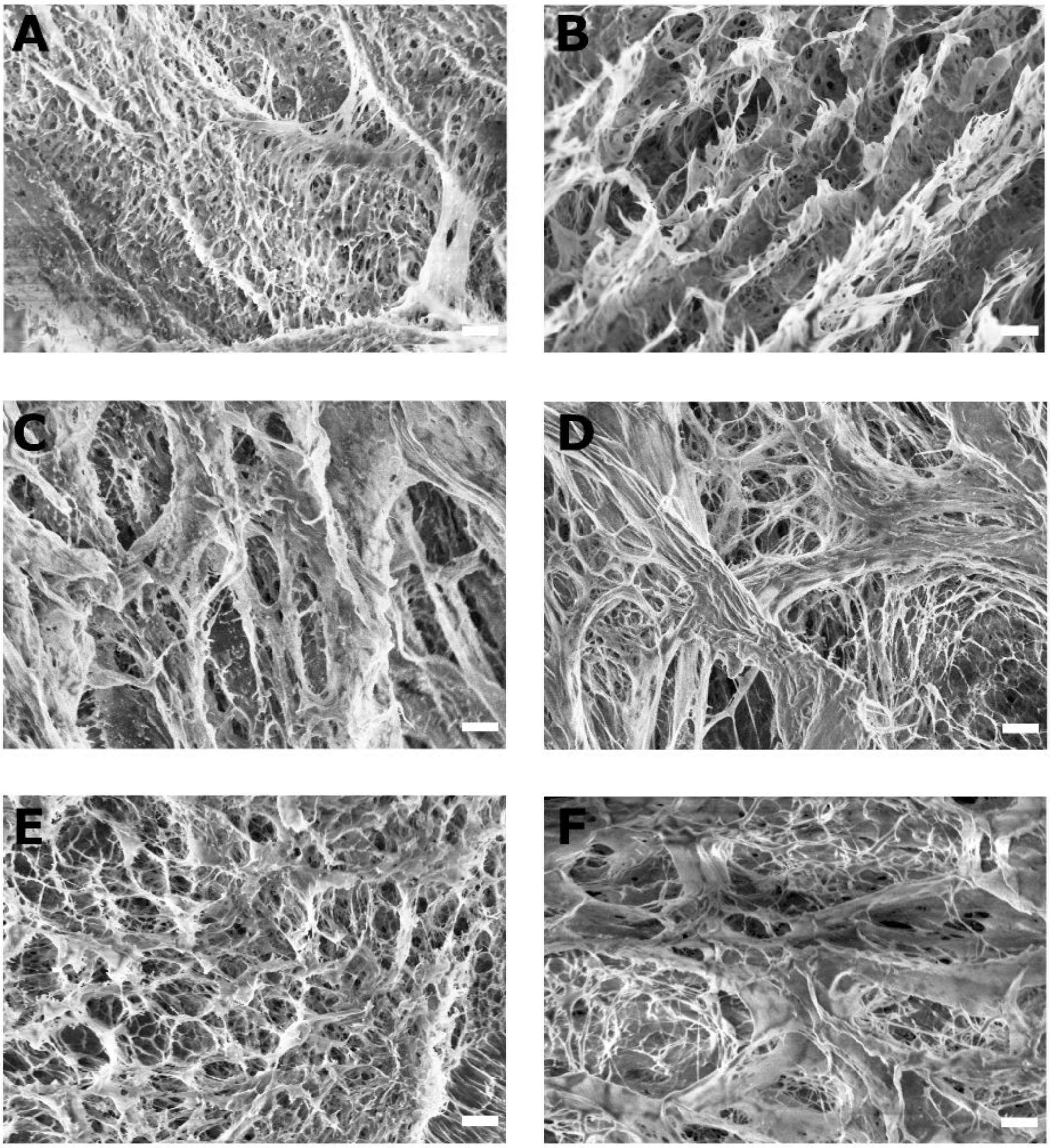

Figure S5. SEM micrographs of A) PN_5; B) PN_12; C) i-PVA_5; D) i-PVA_12; E) i-PVP_5; F) i-PVP_12. Defects (broken gel strands and pitched strand surface) are clearly visible in i-PVA_5. Scalebar: 1 µm.

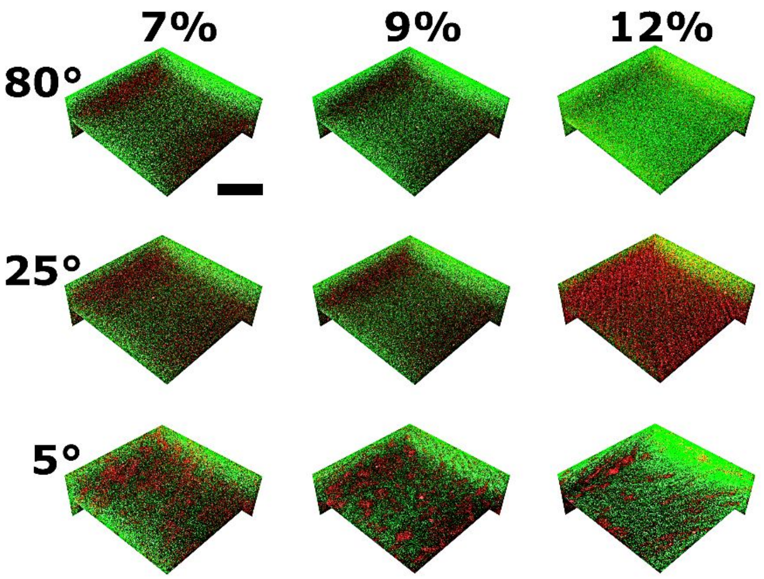

Figure S6. Phase behavior of i-PVP solutions containing total polymer concentrations of 7, 9 and 12 % w/w (H-PVA-PVP mixtures with ratio 3:1). Solutions are homogeneous, until they are cooled to 5°C: at this temperature, PVP blobs are evident. Scalebar: 50 µm.

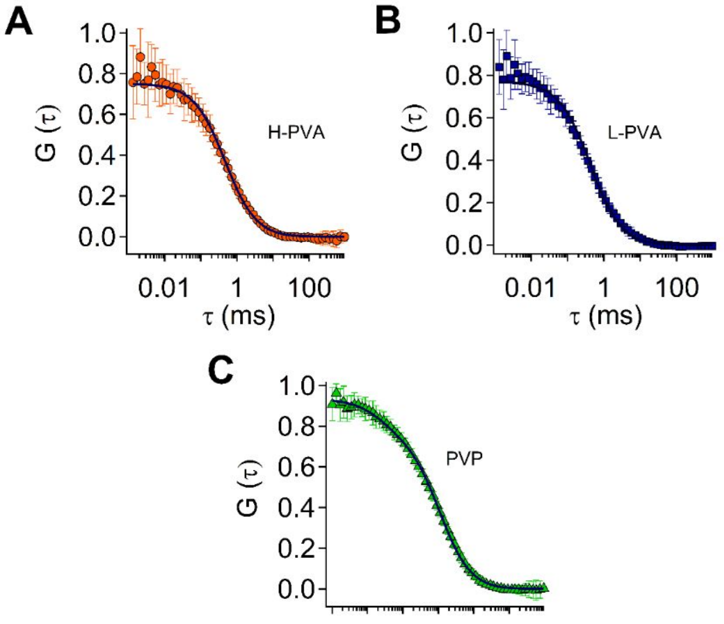

Figure S7. FCS curves of the three labeled polymers in diluted aqueous solution: (A) H-PVA, (B) L-PVA, (C) PVP. The diffusion coefficients obtained by curves fitting were used to calculate the hydrodynamic radius.

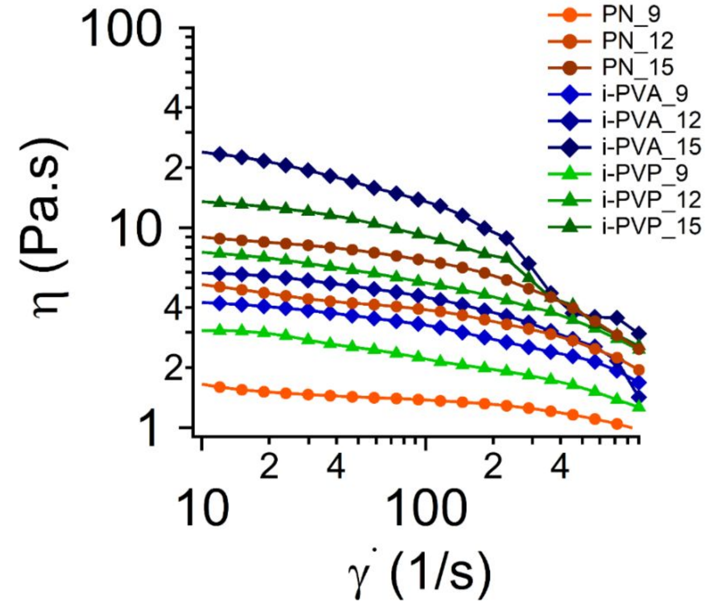

Figure S8. Shear thinning behavior of PN (orange markers), i-PVA (blue markers) and i-PVP (green markers) pre-gel solutions. At high H-PVA concentrations (X = 15) i-PVA solution is the most viscous and displays the stronger shear-thinning behavior, suggesting stronger H-PVA self-interactions.

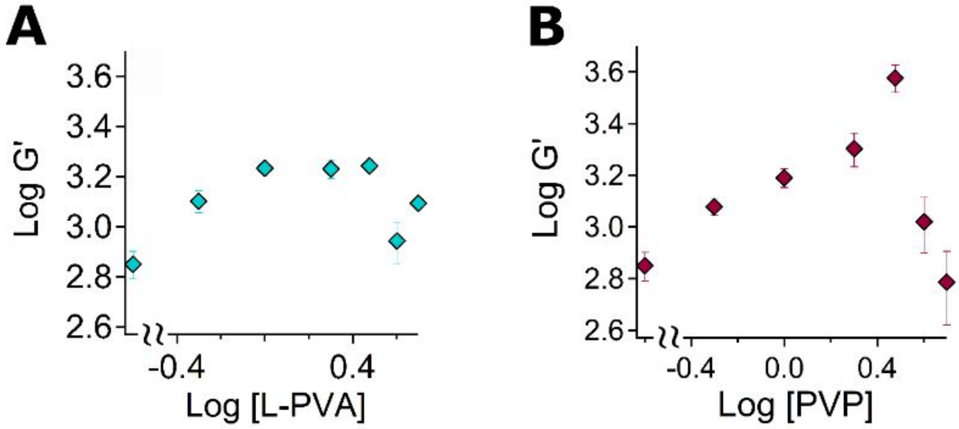

Figure S9. Log-log plots of the storage modulus, G' (1 Hz), versus the porogen polymer concentrations in i(Y)-PVA and i(Y)-PVP gels. A) Effect of increasing concentrations of L-PVA (Y = 0.5, 1, 2, 3, 4, 5 % w/v) on a 9% w/v H-PVA solution. B) Effect of increasing concentrations of PVP (Y = 0.5, 1, 2, 3, 4, 5 % w/v) on a 9% w/v H-PVA solution. The first point in both series represents a pure H-PVA gel (i.e., it is the G' of PN_9 sample).

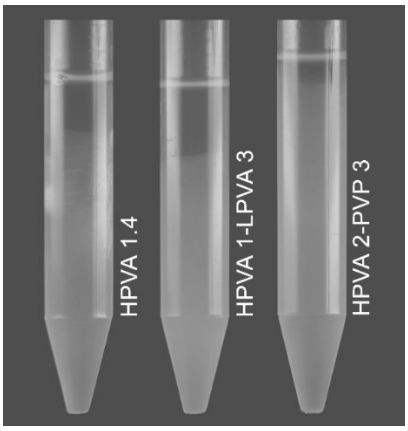

Figure S10. Gel points for PN, i-PVA and i-PVP series: H-PVA formed a gel at 1.4 % w/v; when mixed with 3% w/v L-PVA, H-PVA forms a gel at 1% w/v, while in presence of PVP at 3% w/v the gel point of H-PVA increases to 2 % w/v. Water on top is due to syneresis.

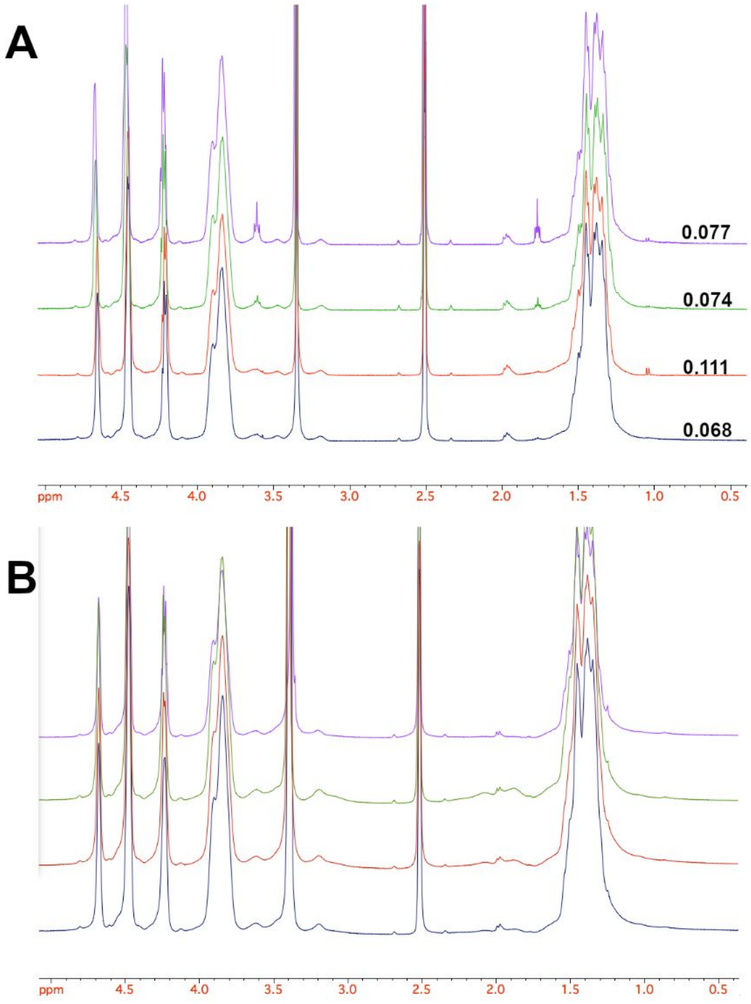

Figure S11. NMR spectra of i(Y)-PVA and i(Y)-PVP gels after washing and storage in water (2 months). A)NMR spectra recorded for i(5)-PVA (violet), i(4)-PVA (green), i(3)-PVA (orange) and i(2)-PVA (blue); the final L-PVA/H-PVA ratio, calculated according to Eq.7, is indicated on the right. B) NMR spectra recorded for i(5)-PVP (violet), i(4)-PVP (green), i(3)-PVP (orange) and i(2)-PVP (blue); in this case only a minor trend is visible, but the amount of residual PVP is negligible.

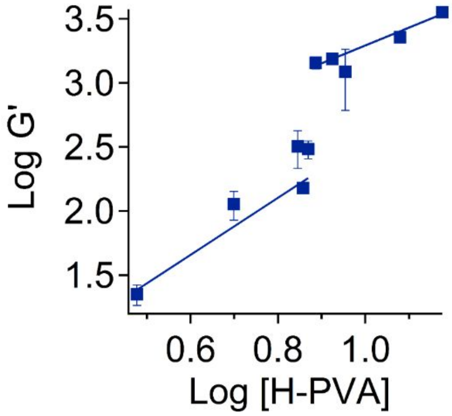

Figure S12. Log-log plots of the storage modulus (G', 1 Hz) versus the concentration of H-PVA in i-PVA series. The slopes of the two curves were used to calculate the fractal dimensions according to JM theory (see Table S3).

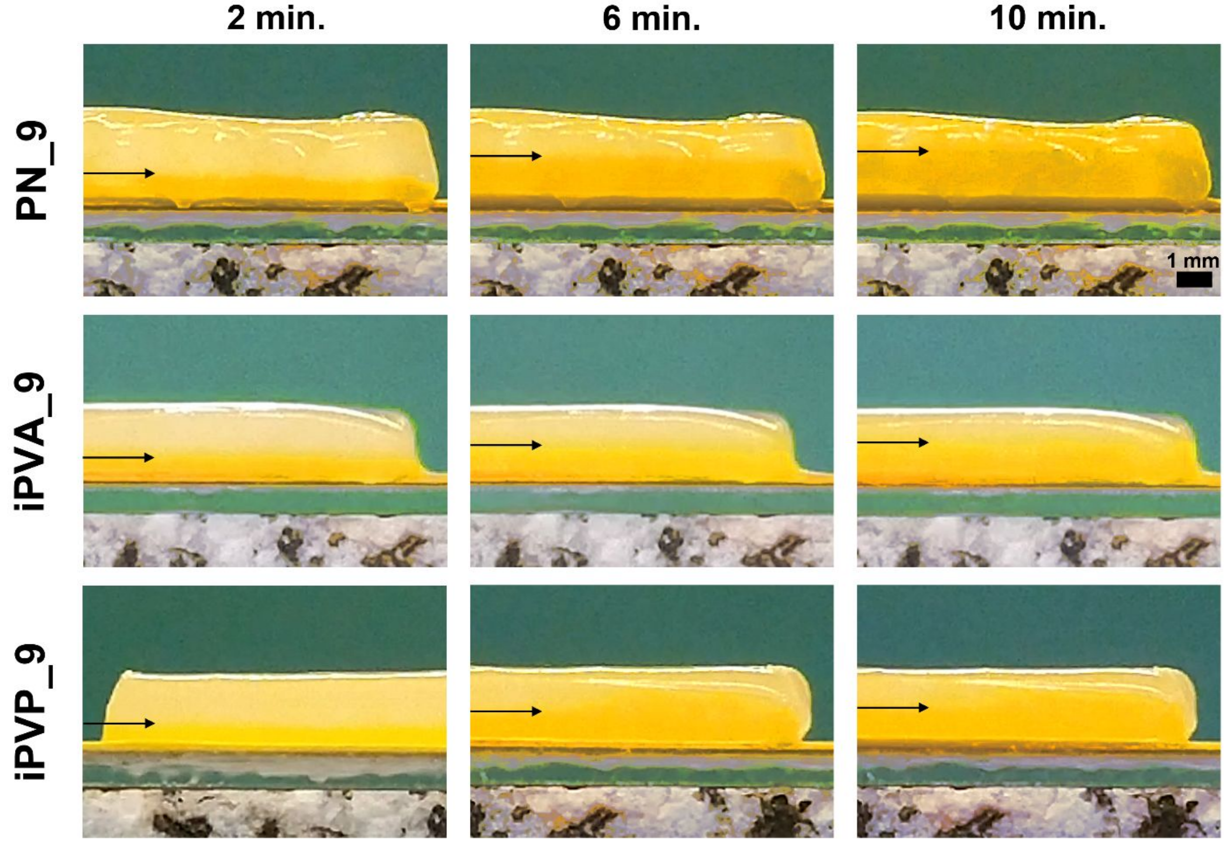

Figure S13. Magnifications of highly contrasted pictures showing the dye uptake of X = 9 gels from cardboards. The height of the solution front was used to construct the graph in fig. 6 A.

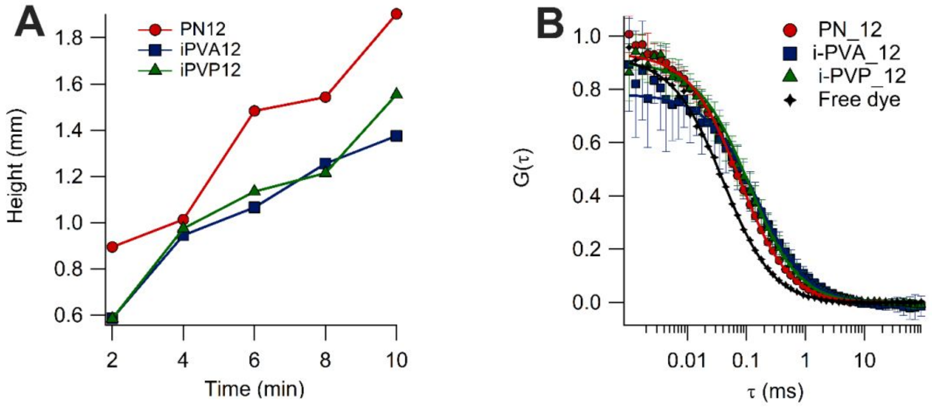

Figure S14. Experiments of dye uptake from a tartrazine-dyed paper sheet and dye diffusion through the gels (tortuosity), for the X=12 samples; (A) Height of the dyed solution inside the networks over a 10 minutes interaction with the dyed paper; (B) FCS curves of a second dye (Alexa Fluor 568, diluted) in a tartrazine concentrated solution (used to dye the paper; black markers), and in the three gels after a 10 minutes interaction with the dyed paper.

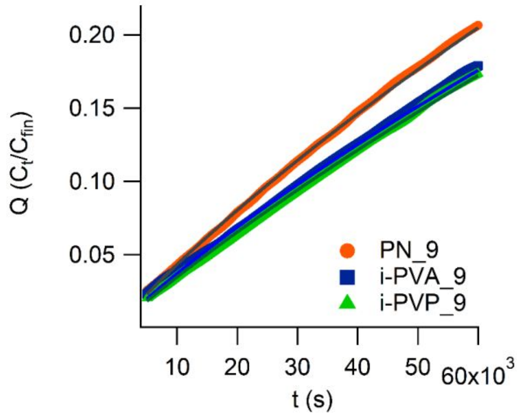

Figure S15. Weibull fitting (solid lines) of kinetic curves describing tartrazine diffusion through X = 9 gels. Experiments were performed through a home-made Franz-type diffusion cell setup.

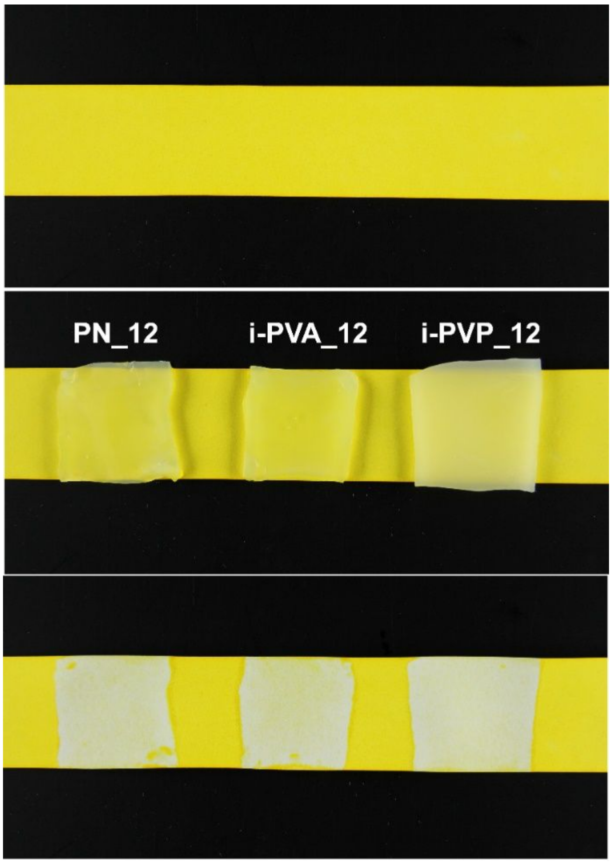

Figure S16. Cleaning performances of X = 12 gels. Top panel: paper sheet imbibed in a tartrazine solution and dried. Central panel, from left to right: PN_12, i-PVA_12, i-PVP_12. Bottom panel: cleaned areas left after a 10 min. contact with the gel sheets; cleaning ability increases with gels tortuosity: PN_12 < i-PVA_12 < i-PVP_12.

Additional Tables.

Table S1. Diffusion coefficients and hydrodynamic radii of H-PVA, L-PVA and PVP in dilute solution, obtained by fitting FCS curves. For PVP, also the diffusion of some free dye (D_2) was detected.

Polymer in dilute solution	D ($\mu m^2/s$)	Hydrodynamic Radius (nm)
**H-PVA**	20 ± 1	12 ± 1
**L-PVA**	26 ± 2	9 ± 1 **
**PVP**	D_1= 10 ± 1 (80%)<br>D_2=420 (for free RBITC)	25 ± 1

**Related to a collapsed conformation, if compared to the values obtained by Budhlall *et al.*[14]

Table S2. Complete SAXS fitting parameters obtained for PN_5-12, i-PVA_5-12 and i-PVP_5-12 cryogels.

Sample	Guinier Scale $I_G(0)$	Lorentz Scale $I_L(0)$	$I_G(0)$/ $I_L(0)$	Correlation Length (nm)	Fractal Dimension (D)	Crystallites Radius R (nm)	Background (Bkg)
**PN_5**	0.3	5.5	0.05	5.7 ± 0.8	2.1 ± 0.1	3.9 ± 0.2	0.06
**PN_7**	0.9	6.9	0.13	5.4 ± 0.8	2.2 ± 0.1	5.0 ± 0.1	0.06
**PN_9**	2.6	11	0.24	5.2 ± 0.8	2.1 ± 0.1	5.1 ± 0.1	0.09
**PN_12**	4.1	14	0.29	4.9 ± 0.4	2.2 ± 0.1	5.2 ± 0.1	0.14
**i-PVA_5**	0.4	8.1	0.05	4.7 ± 0.1	2.1 ± 0.1	5.3 ± 0.4	0.09
**i-PVA_7**	2.2	14	0.16	4.6 ± 0.1	2.2 ± 0.1	5.1 ± 0.2	0.11
**i-PVA_9**	2.0	12	0.17	3.4 ± 0.1	2.6 ± 0.1	6.2 ± 0.2	0.13
**i-PVA_12**	4.8	15	0.32	3.3 ± 0.1	2.7 ± 0.1	5.9 ± 0.2	0.12
**i-PVP_5**	1.4	11	0.13	3.6 ± 0.1	2.5 ± 0.1	5.8 ± 0.2	0.10
**i-PVP_7**	3.1	13	0.24	3.7 ± 0.1	2.4 ± 0.1	5.4 ± 0.1	0.07
**i-PVP_9**	3.3	14	0.24	3.7 ± 0.1	2.5 ± 0.1	5.2 ± 0.1	0.13
**i-PVP_12**	3.2	18	0.18	4.3 ± 0.1	2.5 ± 0.1	4.8 ± 0.1	0.16

Table S3. Values of the exponent n obtained for the gels after thawing, AT (see fig. 6 A, C, E), effective concentration of polymer constituting the network. The exponents, obtained from the

Jones-Marques theory (JM) and the relative surface fractal dimension of fibers, are listed for the i-PVA series.

Series	n (AT)	n (AW)	n (JM model)	D_F
PN	1.69 ± 0.06	3.3 ± 0.5	-	-
i-PVA	1.7 ± 0.1	5.6 ± 0.4	2.2 ± 0.2	1.1
	1.3 ± 0.1	0.7 ± 0.4	1.4 ± 0.1	0.9
i-PVP	1.96 ± 0.02	2.3 ± 0.5	-	-

Table S4. Fitting parameters of diffusion kinetic curves, obtained from Ritger-Peppas (Eq. 9) and Weibull (Eq. 10) equations.

**Sample**	**Ritger-Peppas**		**Weibull**	
	**K (x 10^{-5})**	**n**	**a (x 10^{-6})**	**b**
**PN_9**	4.6 ± 0.2	0.77 ± 0.01	9.5 ± 0.1	0.92 ± 0.01
**i-PVA_9**	2.7 ± 0.2	0.80 ± 0.01	8.7 ± 0.1	0.91 ± 0.01
**i-PVP_9**	2.3 ± 0.1	0.81 ± 0.01	6.5 ± 0.1	0.93 ± 0.01

Table S5. Diffusion coefficients of Alexa Fluor 568 in a tartrazine aqueous solution and through the networks, and corresponding τ^2/ε, *i.e.* D_{Sol}/D_{Gel}, calculated according to eq. 11 in Supporting Information File.

Sample	D ($\mu m^2/s$)	τ^2/ε (D_{Sol}/D_{Gel})
**Tartrazine aqueous solution (2.5% w/w)**	278 ± 14	--
**PN_9**	263 ± 16	1.0
**i-PVA_9**	127 ± 8	2.2
**i-PVP_9**	99 ± 10	2.8
**PN_12**	147 ± 3	1.9
**i-PVA_12**	74 ± 6	3.6
**i-PVP_12**	102 ± 7	2.7

Table S6. Water release and dye removal of gels on carboards dyed with tartrazine. Water release was measured after a 10 minutes contact (the same time of the dye removal experiments) with standard deviations, obtained by repeating the experiment three times. The dye removal was quantified in greyscale images: as the grey scale ranges between 0 (black) and 255 (white), higher values in the table indicate a higher dye removal. Values and standard deviations were obtained by considering the intensity in each pixel of 400 x 400 $pixels^2$ areas, centered inside the cleaned areas.

Sample	Water release (mg/cm^2)	Greyscale Intensity in cleaned areas
**PN_9**	20 ± 1	184 ± 5
**i-PVA_9**	19 ± 1	189 ± 6
**i-PVP_9**	18 ± 1	193 ± 4
**PN_12**	15 ± 1	191 ± 3
**i-PVA_12**	15 ± 1	194 ± 5
**i-PVP_12**	14 ± 1	200 ± 3

References

(1) Levitz, P. Toolbox for 3D Imaging and Modeling of Porous Media: Relationship with Transport Properties. *Cem. Concr. Res.* **2007**, *37* (3), 351–359. https://doi.org/10.1016/j.cemconres.2006.08.004.

(2) MacIver, M. R.; Pawlik, M. Analysis of In Situ Microscopy Images of Flocculated Sediment Volumes. *Chem. Eng. Technol.* **2017**, *40* (12), 2305–2313. https://doi.org/10.1002/ceat.201600523.

(3) Blanton, T. N.; Rajeswaran, M.; Stephens, P. W.; Whitcomb, D. R.; Misture, S. T.; Kaduk, J. A. Crystal Structure Determination of the Silver Carboxylate Dimer [Ag(O2C22H43)]2, Silver Behenate, Using Powder X-Ray Diffraction Methods. *Powder Diffr.* **2011**, *26* (4), 313–320. https://doi.org/10.1154/1.3661981.

(4) Doucet, M.; Cho, J. H.; Alina, G.; Bakker, J.; Bouwman, W.; Butler, P.; Campbell, K.; Gonzales, M.; Heenan, R.; Jackson, A.; Juhas, P.; King, S.; Kienzle, P.; Krzywon, J.; Markvardsen, A.; Nielsen, T.; O'Driscoll, L.; Potrzebowski, W.; Ferraz Leal, R.; Richter, T.; Rozycko, P.; Snow, T.; Washington, A. SasView Version 4.1.2, 2017. https://doi.org/10.5281/zenodo.825675.

(5) Mallam, S.; Horkay, F.; Hecht, A. M.; Rennie, A. R.; Geissler, E. *Microscopic and macroscopic thermodynamic observations in swollen poly(dimethylsiloxane) networks*. https://doi.org/10.1021/ma00002a031.

(6) Shibayama, M.; Tanaka, T.; Han, C. C. Small Angle Neutron Scattering Study on Poly(N-isopropyl Acrylamide) Gels near Their Volume-phase Transition Temperature. *J. Chem. Phys.* **1992**, *97* (9), 6829–6841. https://doi.org/10.1063/1.463636.

(7) Montis, C.; Maiolo, D.; Alessandri, I.; Bergese, P.; Berti, D. Interaction of Nanoparticles with Lipid Membranes: A Multiscale Perspective. *Nanoscale* **2014**, *6* (12), 6452–6457. https://doi.org/10.1039/C4NR00838C.

(8) Koone, N.; Shao, Y.; Zerda, T. W. Diffusion of Simple Liquids in Porous Sol-Gel Glass. *J. Phys. Chem.* **1995**, *99* (46), 16976–16981. https://doi.org/10.1021/j100046a025.

(9) Tjaden, B.; Brett, D. J. L.; Shearing, P. R. Tortuosity in Electrochemical Devices: A Review of Calculation Approaches. *Int. Mater. Rev.* **2018**, *63* (2), 47–67. https://doi.org/10.1080/09506608.2016.1249995.

(10) Briscoe, B.; Luckham, P.; Zhu, S. The Effects of Hydrogen Bonding upon the Viscosity of Aqueous Poly(Vinyl Alcohol) Solutions. *Polymer* **2000**, *41* (10), 3851–3860. https://doi.org/10.1016/S0032-3861(99)00550-9.

(11) Marin, E.; Rojas, J.; Ciro, Y. A Review of Polyvinyl Alcohol Derivatives: Promising Materials for Pharmaceutical and Biomedical Applications. *Afr. J. Pharm. Pharmacol.* **2014**. https://doi.org/10.5897/AJPP2013.3906.

(12) Mastrangelo, R.; Chelazzi, D.; Poggi, G.; Fratini, E.; Buemi, L. P.; Petruzzellis, M. L.; Baglioni, P. Twin-Chain Polymer Hydrogels Based on Poly(Vinyl Alcohol) as New Advanced Tool for the Cleaning of Modern and Contemporary Art. *Proc. Natl. Acad. Sci.* **2020**. https://doi.org/10.1073/pnas.1911811117.

(13) Lewandowska, K.; Staszewska, D. U.; Bohdanecký, M. The Huggins Viscosity Coefficient of Aqueous Solution of Poly(Vinyl Alcohol). *Eur. Polym. J.* **2001**, *37* (1), 25–32. https://doi.org/10.1016/S0014-3057(00)00074-4.

(14) Budhlall, B. M.; Landfester, K.; Sudol, E. D.; Dimonie, V. L.; Klein, A.; El-Aasser, M. S. Characterization of Partially Hydrolyzed Poly(Vinyl Alcohol). Effect of Poly(Vinyl Alcohol) Molecular Architecture on Aqueous Phase Conformation. *Macromolecules* **2003**, *36* (25), 9477–9484. https://doi.org/10.1021/ma030027d.

(15) Gao, H.; He, J.; Yang, R.; Yang, L. Characteristic Rheological Features of High Concentration PVA Solutions in Water with Different Degrees of Polymerization. *J. Appl. Polym. Sci.* **2010**, *116* (5), 2734–2741. https://doi.org/10.1002/app.31900.

(16) Gao, H.-W.; Yang, R.-J.; He, J.-Y.; Yang, L. Rheological Behaviors of PVA/H2O Solutions of High-Polymer Concentration. *J. Appl. Polym. Sci.* **2010**, *116* (3), 1459–1466. https://doi.org/10.1002/app.31677.

(17) Nishio, Y.; Haratani, T.; Takahashi, T. Miscibility and Orientation Behavior of Poly(Vinyl Alcohol) / Poly(Vinyl Pyrrolidone) Blends. *J. Polym. Sci. Part B Polym. Phys.* **1990**, *28* (3), 355–376. https://doi.org/10.1002/polb.1990.090280308.

(18) Ping, Z.-H.; Nguyen, Q. T.; Néel, J. Investigations of Poly(Vinyl Alcohol)/Poly(N-Vinyl-2-Pyrrolidone) Blends, 2. Influence of the Molecular Weights of the Polymer Components on Crystallization. *Makromol. Chem.* **1990**, *191* (1), 185–198. https://doi.org/10.1002/macp.1990.021910115.

(19) Lewandowska, K. The Miscibility of Poly(Vinyl Alcohol)/Poly(N-Vinylpyrrolidone) Blends Investigated in Dilute Solutions and Solids. *Eur. Polym. J.* **2005**, *41* (1), 55–64. https://doi.org/10.1016/j.eurpolymj.2004.08.016.

(20) Abou_Taleb, M. H. Thermal and Spectroscopic Studies of Poly(N-Vinyl Pyrrolidone)/Poly(Vinyl Alcohol) Blend Films. *J. Appl. Polym. Sci.* **2009**, *114* (2), 1202–1207. https://doi.org/10.1002/app.30082.

(21) Bernal, A.; Kuritka, I.; Saha, P. Preparation and Characterization of Poly(Vinyl Alcohol)-Poly(Vinyl Pyrrolidone) Blend: A Biomaterial with Latent Medical Applications. *J. Appl. Polym. Sci.* **2013**, *127* (5), 3560–3568. https://doi.org/10.1002/app.37723.

(22) Bercea, M.; Morariu, S.; Teodorescu, M. Rheological Investigation of Poly(Vinyl Alcohol)/Poly(N-Vinyl Pyrrolidone) Mixtures in Aqueous Solution and Hydrogel State. *J. Polym. Res.* **2016**, *23* (7), 142. https://doi.org/10.1007/s10965-016-1040-3.

(23) Teodorescu, M.; Bercea, M.; Morariu, S. Miscibility Study on Polymer Mixtures in Dilute Solution. *Colloids Surf. Physicochem. Eng. Asp.* **2018**, *559*, 325–333. https://doi.org/10.1016/j.colsurfa.2018.09.062.

(24) Boonsuk, P.; Kaewtatip, K.; Chantarak, S.; Kelarakis, A.; Chaibundit, C. Super-Tough Biodegradable Poly(Vinyl Alcohol)/Poly(Vinyl Pyrrolidone) Blends Plasticized by Glycerol and Sorbitol. *J. Appl. Polym. Sci.* **2018**, *135* (26), 46406. https://doi.org/10.1002/app.46406.

(25) Zaslavsky, B. Y. *Aqueous Two-Phase Partitioning: Physical Chemistry and Bioanalytical Applications*; CRC Press, 1994.

(26) Guettari, M.; Belaidi, A.; Abel, S.; Tajouri, T. Polyvinylpyrrolidone Behavior in Water/Ethanol Mixed Solvents: Comparison of Modeling Predictions with Experimental Results. *J. Solut. Chem.* **2017**, *46* (7), 1404–1417. https://doi.org/10.1007/s10953-017-0649-0.

(27) Shibayama, M.; Kurokawa, H.; Nomura, S.; Muthukumar, M.; Stein, R. S.; Roy, S. Small-Angle Neutron Scattering from Poly(Vinyl Alcohol)-Borate Gels. *Polymer* **1992**, *33* (14), 2883–2890. https://doi.org/10.1016/0032-3861(92)90072-5.

(28) Ramzi, M.; Rochas, C.; Guenet, J.-M. Structure−Properties Relation for Agarose Thermoreversible Gels in Binary Solvents. *Macromolecules* **1998**, *31* (18), 6106–6111. https://doi.org/10.1021/ma9801220.

(29) Guenet, J.-M. Structure versus Rheological Properties in Fibrillar Thermoreversible Gels from Polymers and Biopolymers. *J. Rheol.* **2000**, *44* (4), 947–960. https://doi.org/10.1122/1.551121.

(30) Hernández, R.; Sarafian, A.; López, D.; Mijangos, C. Viscoelastic Properties of Poly(Vinyl Alcohol) Hydrogels and Ferrogels Obtained through Freezing–Thawing Cycles. *Polymer* **2004**, *45* (16), 5543–5549. https://doi.org/10.1016/j.polymer.2004.05.061.

(31) Artyukhov, A. A.; Shtilman, M. I.; Kuskov, A. N.; Pashkova, L. I.; Tsatsakis, A. M.; Rizos, A. K. Polyvinyl Alcohol Cross-Linked Macroporous Polymeric Hydrogels: Structure Formation and Regularity Investigation. *J. Non-Cryst. Solids* **2011**, *357* (2), 700–706. https://doi.org/10.1016/j.jnoncrysol.2010.06.038.

(32) Hernandez, R.; Lopez, D.; Mijangos, C.; Guenet, J.-M. A Reappraisal of the 'Thermoreversible' Gelation of Aqueous Poly(Vinyl Alcohol) Solutions through Freezing–Thawing Cycles. *Polymer* **2002**, *43* (21), 5661–5663. https://doi.org/10.1016/S0032-3861(02)00470-6.
